# Nicotinic receptor components of amyloid beta 42 proteome regulation in human neural cells

**DOI:** 10.1371/journal.pone.0270479

**Published:** 2022-08-12

**Authors:** Patricia Sinclair, Nadine Kabbani

**Affiliations:** 1 Interdisciplinary Program in Neuroscience, George Mason University, Fairfax, VA, United States of America; 2 School of System Biology, George Mason University, Fairfax, VA, United States of America; National Center of Neurology and Psychiatry (NCNP), JAPAN

## Abstract

Alzheimer’s disease (AD) is associated with chronic neurodegeneration often accompanied by elevated levels of the neurotoxic peptide amyloid-beta 1–42 (Aβ_42_) in the brain. Studies show that extracellular Aβ_42_ binds to various cell surface receptors including the human α7 nicotinic acetylcholine receptor (nAChR) and activates pathways of neurotoxicity leading to cell death. The α7 nAChR is thus considered a promising drug target for therapy against neurodegenerative disease such as AD. In this study, we use mass spectrometry-based label-free precursor ion quantification to identify proteins and pathways that are changed by a 72-hour treatment with Aβ_42_ or Aβ_42_ in the presence of the α7 nAChR blocker, α-bungarotoxin (Bgtx) in the human neuroblastoma SH-SY5Y cell line. Bioinformatic gene ontology enrichment analysis was used to identify and characterize proteins and pathways altered by Aβ_42_ presentation. The results support evidence on the involvement of mitochondrial proteins in Aβ_42_ responses and define potential mechanisms of α7 nAChR mediated amyloid toxicity. These findings can inform pharmacological strategies for drug design and treatment against amyloid disease.

## Introduction

Extracellular amyloid plaques and intracellular tangles of hyperphosphorylated tau are physiological hallmarks of Alzheimer’s disease (AD) used to confirm diagnosis during post-mortem examination of brain tissue [1]. While much remains unknown about the etiology and cellular pathology underlying AD, insight from genetic predisposing factors suggest that AD may arise from variability in lipid and protein processing within neural cells. In particular, mutations in genes for amyloid precursor protein (APP) and its processing favor increased generation of the pathogenic amyloid-beta 1–42 (Aβ_42_) peptide and confer susceptibility to early onset AD [2]. The self-assembly of Aβ_42_, which is seen both *in vivo* and *in vitro*, leads to the formation of higher order amyloid structures (e.g. oligomers) which appear to drive disrupted signaling in cells and brain tissue [3]. Studies show that Aβ_42_ can critically drive membrane calcium signaling, disrupt intracellular protein trafficking and degradation, and increase mitochondrial oxidative stress in various types of neural cells [4].

AD degeneration appears early in the cholinergic neurons of the basal forebrain and impacts projections to regions such as the entorhinal cortex [5]. Various components of the cholinergic system, including nicotinic acetylcholine receptors (nAChR) are expressed in these basal forebrain neurons and have been implicated in amyloid neurotoxicity [6, 7]. Studies show that Aβ_42_ can directly bind the α7 nAChR within the orthosteric ligand binding site thus altering calcium entry into neurons [8–10]. In addition to calcium signaling, α7 nAChRs are known to activate several downstream signaling pathways and regulate cytoskeletal and mitochondrial activity [9, 10]. In SH-SY5Y cells oligomeric Aβ_42_ peptide was found to mediate a robust inhibition of ERK phosphorylation induced by choline activation of α7 nAChRs [11]. Targeting the α7 nAChR has been suggested as an important strategy for drug development against AD and other neuro-disorders [12, 13].

In recent work we profiled responses to Aβ_42_ within the proteome of nerve growth factor (NGF) differentiated pheochromocytoma 12 (PC12) cells using tandem mass spectrometry-based label-free quantitative analysis coupled to bioinformatics [14]. Here, we extend this approach to determine the impact of Aβ_42_ in the human neuroblastoma SH-SY5Y cell line, which is an established model for the study of amyloid processing [15, 16]. We begin to explore the involvement of α7 nAChRs in Aβ_42_ neurotoxicity by comparing proteome responses in cells treated with Aβ_42_ in the presence or absence of the α7 nAChR blocker Bgtx.

## Methods and materials

### Cell culture and drug treatment

Human neuroblastoma cells SH-SY5Y cells (ATCC® CRL-2266™) were grown in DMEM (Gibco 11995065) supplemented with 10% fetal bovine serum (FBS) and 1% pen/strep in T75 cell culture treated flasks at 37°C and 5% CO2. Passage 10 cells grown to 70% confluence were treated with 100 nM Aβ_42_ (Bachem, H-6466) or its reverse, amyloid-beta 42–1, (Aβ_rev_) (Bachem, H-3976) prepared as described in Arora et al. [17] in the absence or presence of 50 nM Bgtx,. Media was changed daily with drug application [18]. Media change alone was performed on the control group. At 72 hours, cells were lysed and proteins solubilized using a 0.1% Triton X-100 buffer (Triton X-100, 1 M Tris HCl, 1.5 M NaCl, 0.25 M EDTA, and 10% glycerol, in the presence of protease inhibitors (Complete Mini, Roche) as described in Nordman et al. [19]. Protein concentration was determined using a Bradford assay.

### Cell viability

We tested viability in SH-SY5Y cultured cells at the end of the 72-hour treatment using the trypan blue method [20]. Cells were counted using a hemocytometer, and percent viable cells were calculated across treatment conditions relative to control.

### Mitochondrial membrane potential

SH-SY5Y cells were plated on 96-well plates (Cellvis, P-96-1-N) coated with 100 μg/ml poly-D-lysine (Millipore, A-003-E) in culture media for 24 hours before amyloid application. At 72 hours, cells were incubated with 50 nM tetramethyl rhodamine, ethyl ester, perchlorate (TMRE) (Thermo Fisher Scientific, Waltham, MA, USA, T669) for 30 minutes then washed with phosphate buffered solution (PBS). TMRE fluorescence was measured using a Zeiss LSM 800 scanning-laser confocal microscope at 561 nm/595 nm excitation/emission settings. Fluorescence intensities for over 20 regions of interest (ROI) per condition were quantified using ImageJ.

### Liquid-chromatography electrospray ionization mass spectrometry

Solubilized proteins samples were treated with acetone on ice for 5 minutes prior to centrifugation to precipitate proteins. The protein pellet was denatured, reduced, and alkylated with 8 M urea, 1 M dithiothreitol, 0.5 M iodoacetamide. Proteins were digested in 2 μl (0.5 μg/μl) trypsin in 500nM ammonium bicarbonate, then incubated at 37°C for 5 h. After desalting with C-18 ZipTips (Millipore), the samples were dehydrated in a SpeedVac for 18 minutes and reconstituted in 0.1% formic acid for a final volume of 20 μl which was used to provide 3 technical replicates for liquid-chromatography electrospray ionization mass spectrometry (LC-ESI MS/MS). LC-ESI MS/MS was performed using an Exploris Orbitrap 480 equipped with an EASY-nLC 1200HPLC system (Thermo Fischer Scientific, Waltham, MA, USA). Peptides were separated using a reverse-phase PepMap RSLC 75 μm i.d by 15 cm long with a 2 μm particle size C18 LC column (Thermo Fisher Scientific, Waltham, MA, USA), eluted with 0.1% formic acid and 80% acetonitrile at a flow rate of 300 nl/min. Following a full scan at 60,000 resolving power from 300 m/z to 1200 m/z, peptides were fragmented by high-energy collision dissociation (HCD) with a normalized collision energy of 28%. EASY-IC filters for internal mass calibration, monoisotopic precursor selection, and dynamic exclusions (20 s) were enabled. Peptide precursor ions with charge states from +2 to + 4 were included.

### Protein quantification and statistical analysis

Proteins were identified by comparing raw MS peptide spectra to the NCBI human protein database using SEQUEST HT search engine within the Proteome Discoverer *v2*.*4* (Thermo Fisher Scientific, Waltham, MA, USA) using the following parameters: mass tolerance for precursor ions = 2 ppm; mass tolerance for fragment ions = 0.05 Da; and cut-off value for the false discovery rate (FDR) in reporting peptide spectrum matches (PSM) to the database = 1%. Peptide abundance ratio was obtained by precursor ion quantification in Proteome Discoverer *v*2.4, using the vehicle control group as the denominator. Abundance ratios with adjusted p-values < 0.05 determined using a one-way analysis of variance (ANOVA) followed by Benjamini-Hochberg post-hoc analyses were considered statistically significant. Proteins were included for further analysis when matches were found in at least 2 of the 3 replicates with a recorded group abundance. Statistical significance of Gene Ontology (GO) pathways analyzed in Database for Annotation, Visualization, and Integrated Discovery (DAVID) was obtained using a Fisher Exact Test (EASE score) followed by Benjamini-Hochberg correction [21, 22].

### GO enrichment analysis

To perform GO enrichment analyses on the two proteomes (“Aβ_42_^P^” and “Aβ_42_/Bgtx ^P^”) the official gene symbols (HUGO Gene Nomenclature Committee (HGNC)) of proteins with statistically significant (adjusted p-value < 0.05) abundance ratios were uploaded to DAVID (December 2021). The “ΔP” proteome was deduced based on at least one of two criteria: 1) When the Aβ_42_ and Bgtx co-treatment condition is found to result in an opposite abundance ratio measure from the Aβ_42_ treatment condition alone; 2) When the Aβ_42_^P^ abundance ratio measure is returned to control in by Aβ_42_+Bgtx co-treatment.


$$↑A\beta_{42}{}^{P}\&↓A\beta_{42}/Bgtx+↓A\beta_{42}{}^{P}\&↑A\beta_{42}/Bgtx+A\beta_{42}{}^{P}(p<0.05)\&A\beta_{42}/Bgtx(p\geq 0.05)$$ (1)

GO terms were considered enriched in the uploaded dataset after the Fisher Exact Test followed by Benjamini post-hoc analysis resulted in an adjusted p-value < 0.05. Data was organized and figures presented using R statistical packages including ggplot2 [23], tidyverse [24], GOPlot [25], and Venn.Diagram [26]. K-clustering analysis and its visualization were made using Python.

## Results

### Identification of an Aβ_42_ responsive proteome in human neural SH-SY5Y cells

**Fig 1:**
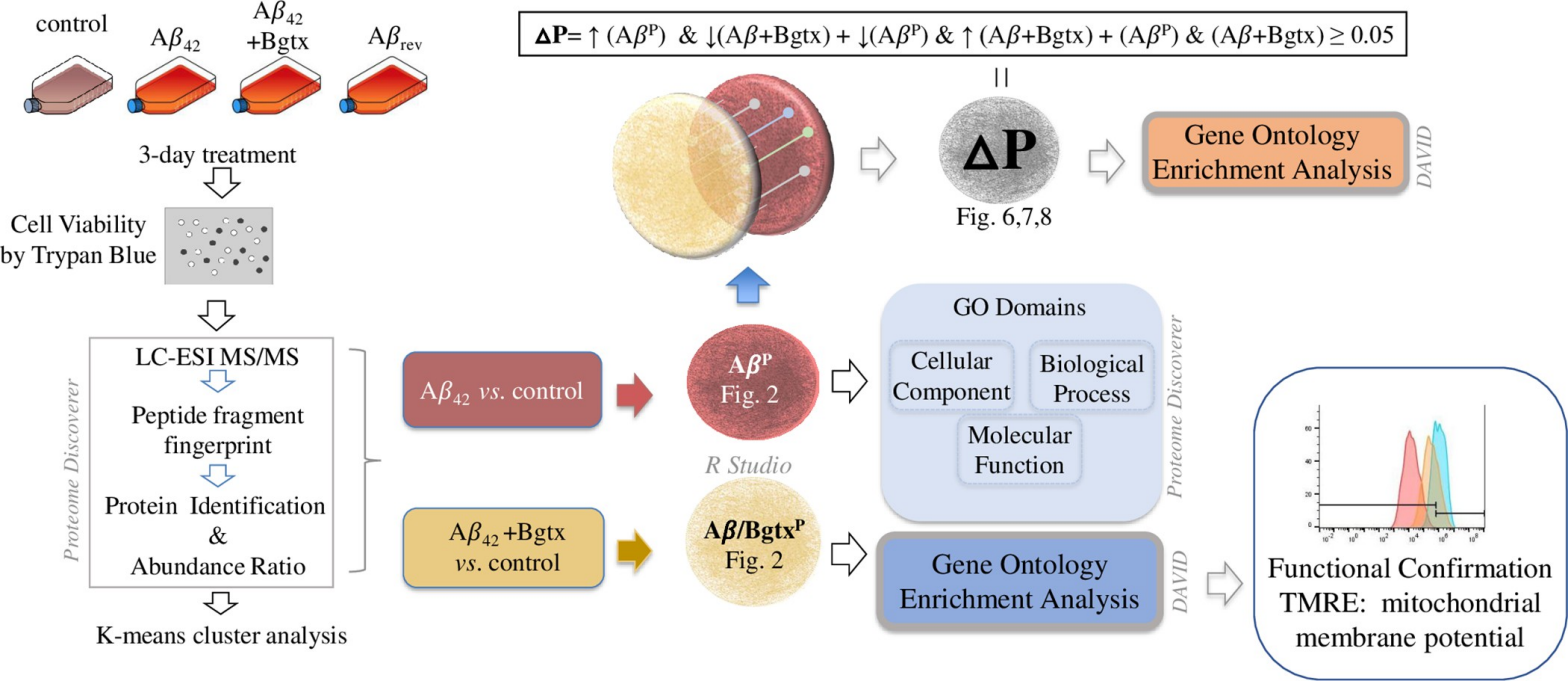
A summary of the experimental design and analyses. A workflow schematic showing the treatment conditions and briefly describing the mass spectrometry and bioinformatic analyses performed.

Aβ_42_ neurotoxicity has been shown to involve multiple signaling pathways through various cellular entry points including cell surface receptors [27]. Recently, we used a proteomic approach to determine the impact of Aβ_42_ exposure in NGF differentiated PC12 cells [14]. We extend this effort to examine proteomic responses to Aβ_42_ in the human SH-SY5Y neuroblastoma cell line, which is widely used as in the study of amyloid toxicity and regulation [28]. SH-SY5Y cells were treated with 100 nM Aβ_42_ prepared in a manner that has been established to favor the formation of pathogenic oligomers [17]. After 72 hours of exposure, cells were processed and analyzed using liquid chromatography electrospray ionization (LC-ESI) tandem mass spectrometry (MS/MS). SH-SY5Y cells grown under the same condition but exposed to the vehicle (media) were used as the experimental control. Whole cell proteomic analysis based on LC-ESI-MS/MS proteomic spectra identification and relative protein quantification was obtained using a label-free precursor ion quantification method [29]. The experimental design and workflow of the study are summarized in Fig 1.

**Fig 2:**
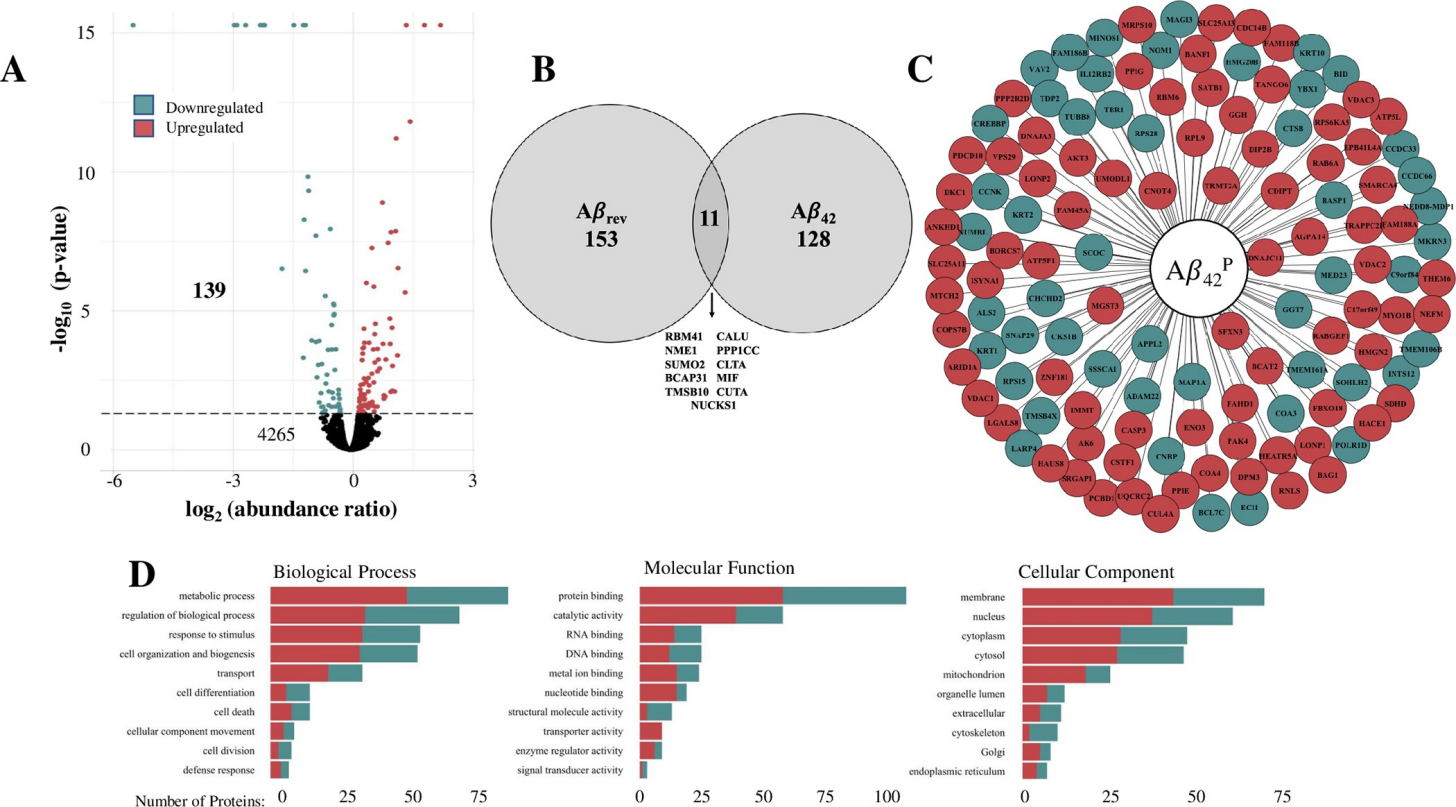
Proteome response to Aβ_42_ treatment. A) The distribution and number of detected proteins within samples of Aβ_42_ treated cells. The horizontal line indicates the threshold for statistical significance (p < 0.05). B) Proteins altered by Aβ_42_ and Aβ_rev_. C) Components of the Aβ_42_^P^ identified by their gene symbols. D) The number of proteins within the Aβ_42_^P^ associated with GO terms.

We examined the potential for toxicity in our treatment condition using trypan blue. Based on a count of viable cells across all treatment conditions (100 nM Aβ_42,_ 100 nM Aβ_42_ + 50 nM Bgtx, or 100 nM Aβ_rev_) relative to control, our 72-hour drug treatment was not associated with cell toxicity (S1 Fig in S1 File). Based on an analysis of the abundance ratio measures in Aβ_42_ treated cells relative to controls we identified significantly altered proteins of the Aβ_42_ proteome (Aβ_42_^P^). MS/MS analysis shows that of the 4706 proteins detected within our SH-SY5Y cell fraction samples, 139 proteins were found to be significantly altered (p value < 0.05) with 58% upregulated and 42% downregulated (Fig 2A) (S1 Table in S1 File). We examined the specificity of Aβ_42_ associated proteomic changes by performing matched experiments in cells using a reverse sequence peptide, Aβ_rev_. As shown in Fig 2B, 72-hour treatment with Aβ_rev_ was found to promote proteomic responses markedly different from Aβ_42_. The full Aβ_rev_ associated proteome is included in S2 Table in S1 File. A comparison of the two proteomes (Aβ_42_ *vs*. Aβ_rev_) indicates 11 common proteins that were excluded from the analysis. The components of the Aβ_42_^P^ are presented in Fig 2C according to their HGNC symbol, and associated Gene Ontology (GO) terms identified using Proteome Discoverer *v*2.4. The results suggest the involvement of metabolic processes, protein binding, and membrane components within Aβ_42_^P^ (Fig 2D).

**Fig 3:**
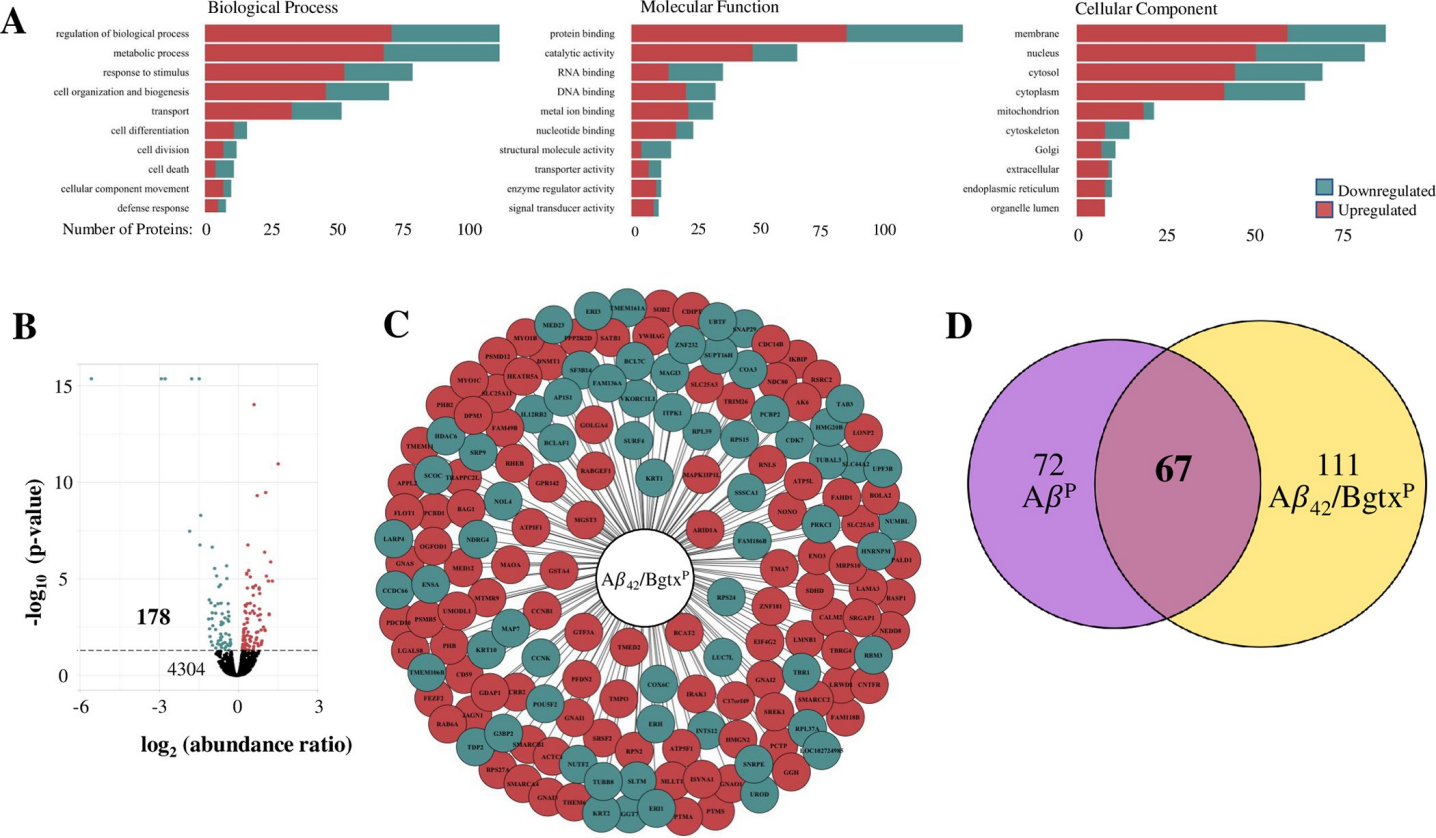
Proteome response to Aβ_42_ + Bgtx co-treatment. A) The number of proteins within the Aβ_42_/Bgtx^P^ associated with GO terms. B) The distribution and number of detected proteins within Aβ_42_ + Bgtx treated cells. The horizontal line indicates the threshold for statistical significance (p < 0.05). C) Components of the Aβ_42_/Bgtx^P^ identified by gene symbols. D) The number of proteins within the Aβ_42_^P^ and Aβ_42_/Bgtx^P^ datasets. 67 proteins are found in both.

Previous articles show that Aβ_42_ binds to the α7 nAChR and drives neurotoxicity [30]. We tested the effect of the α7 nAChR antagonist Bgtx on Aβ_42_ associated proteome modification within SH-SY5Y cells. In these experiments, cells were treated with 100 nM Aβ_42_ in the presence of 50 nM Bgtx for 72 hours followed by MS/MS analysis to identify the Aβ_42_ + Bgtx proteome (Aβ_42_/Bgtx^P^). As shown in Fig 3B and 3C, proteomic analysis indicates 178 significantly altered proteins within Aβ_42_/Bgtx^P^ (S3 Table in S1 File). This proteome consists of 61% upregulated and 29% downregulated proteins in Fig 3B. GO analysis highlights the involvement of metabolic processes, protein binding, and membrane components within Aβ_42_/Bgtx^P^ (Fig 3A) similar to Aβ_42_^P^. A comparison of the Aβ_42_^P^ and Aβ_42_/Bgtx^P^ datasets indicates the presence of 67 (27%) common proteins (Fig 3D).

**Fig 4:**
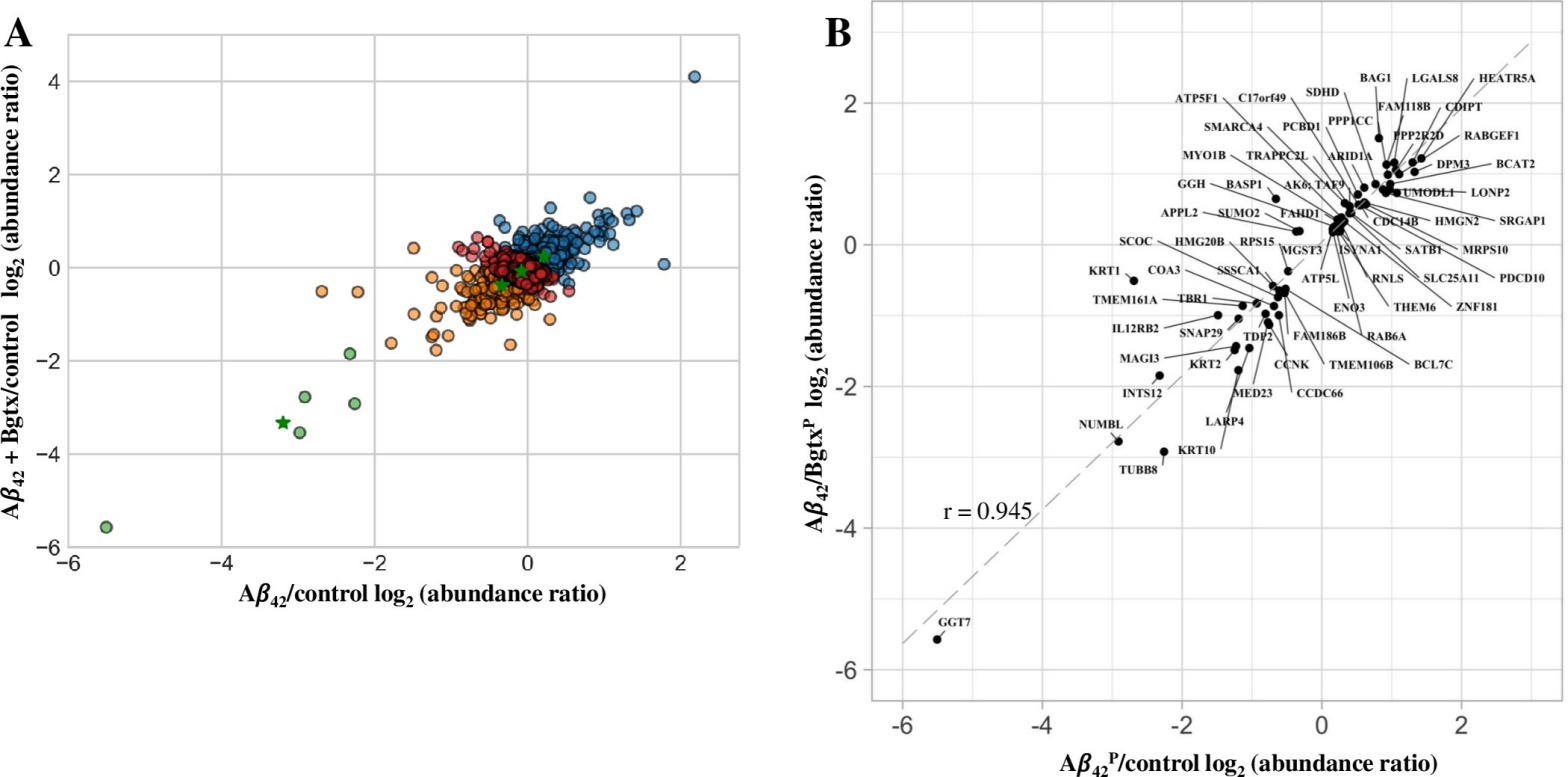
Clustering and correlation analysis of the proteomes. A) A K-means cluster analysis of detected proteins across both Aβ_42_ and Aβ_42_ + Bgtx treatment conditions. The analysis indicates 4 cluster means (green stars). B) Scatterplot and correlation analysis of the log_2_ transformed abundance ratio measures for individual proteins within Aβ_42_^P^ and Aβ_42_/Bgtx^P^.

We performed K-means clustering analysis on proteins detected across treatment conditions (Fig 1). As shown in Fig 4A, a K-means cluster analysis shows log-transformed abundance ratios in the Aβ_42_ alone relative to Aβ_42_ + Bgtx treatment, with 4 cluster means indicated across data points. We then examined the extent of similarity in protein change within Aβ_42_^P^ and Aβ_42_/Bgtx^P^ by plotting the log transformed abundance ratio for each of the two proteomes and then determining a correlation coefficient. As shown in Fig 4B, a strong correlation (r = 0.945) was seen between Aβ_42_^P^ and Aβ_42_/Bgtx^P^ suggesting that most proteins are similarly impacted across both datasets.

### GO analysis of amyloid and nicotinic receptor proteomic mechanisms

**Fig 5:**
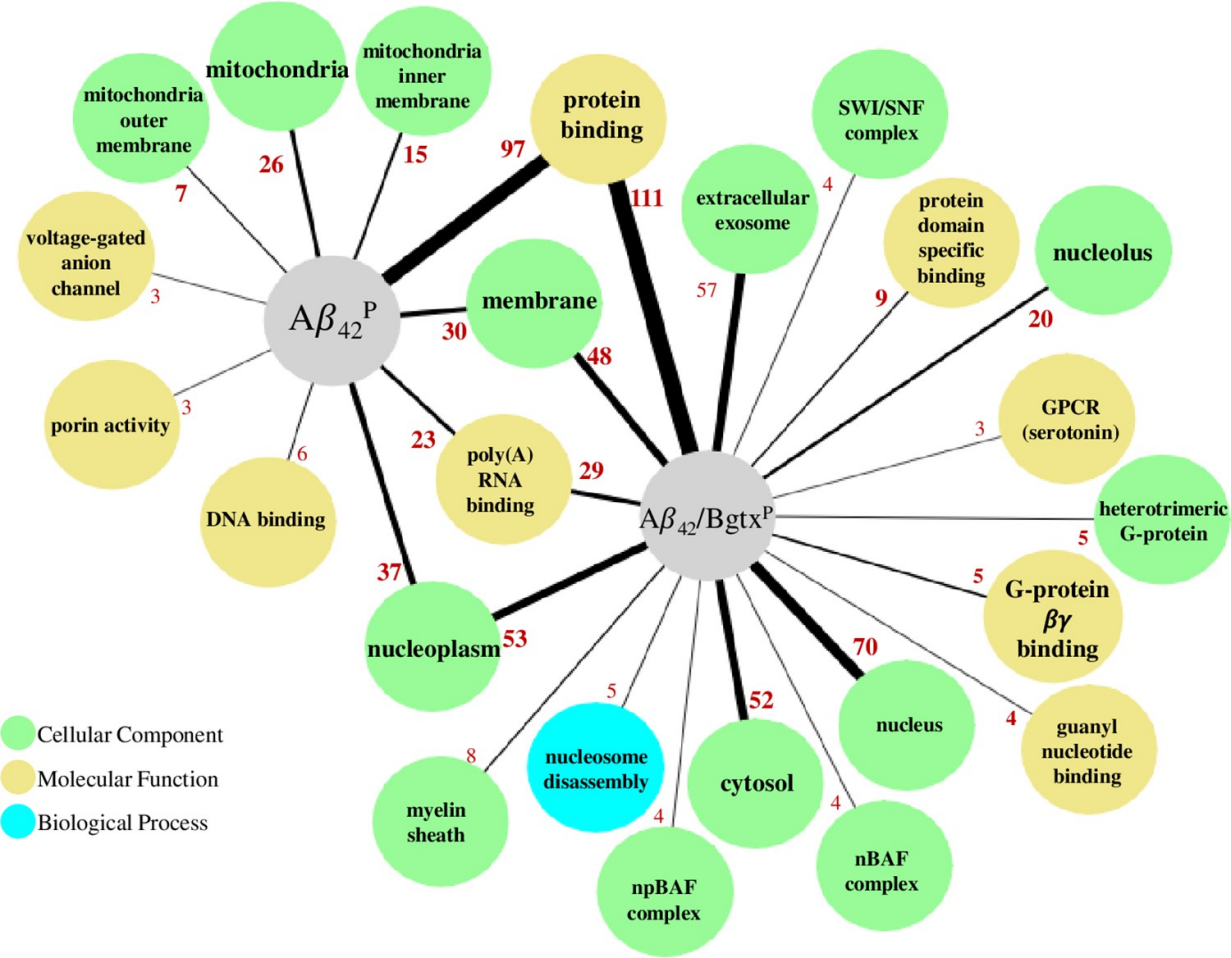
DAVID enrichment analysis of Aβ42P and Aβ/Bgtx^P^. GO terms associated with Aβ_42_^P^ and Aβ_42_/Bgtx^P^. The number of proteins associated with each GO term are indicated by edge width and red lettering.

Important changes in cell growth and function are driven by dynamic adaptations within gene to protein regulatory networks [31, 32]. These changes can be examined qualitatively and quantitatively using various “omic” methods in conjunction with bioinformatic analysis tools [33, 34]. To determine differences within proteomes, we performed enrichment analyses in DAVID on Aβ_42_^P^ and Aβ_42_/Bgtx^P^ (December 2021). As shown in Fig 5, both Aβ_42_^P^ and Aβ_42_/Bgtx^P^ appear to share key GO terms, while also distinctly associated with varied GO components. For example, mitochondria and mitochondrial processes both appear as GO terms in the DAVID enrichment analysis in Aβ_42_^P^, yet guanine nucleotide signaling (e.g., heterotrimeric G proteins) *only* appears in the Aβ_42_/Bgtx^P^ analysis. Similarly, cytosolic and nuclear proteins feature in Aβ_42_/Bgtx^P^ but are not identified within the Aβ_42_^P^. Within Aβ_42_^P^ and Aβ_42_/Bgtx^P^, protein binding, poly(A) RNA binding, nucleoplasm, and membrane are similarly enriched.

**Fig 6:**
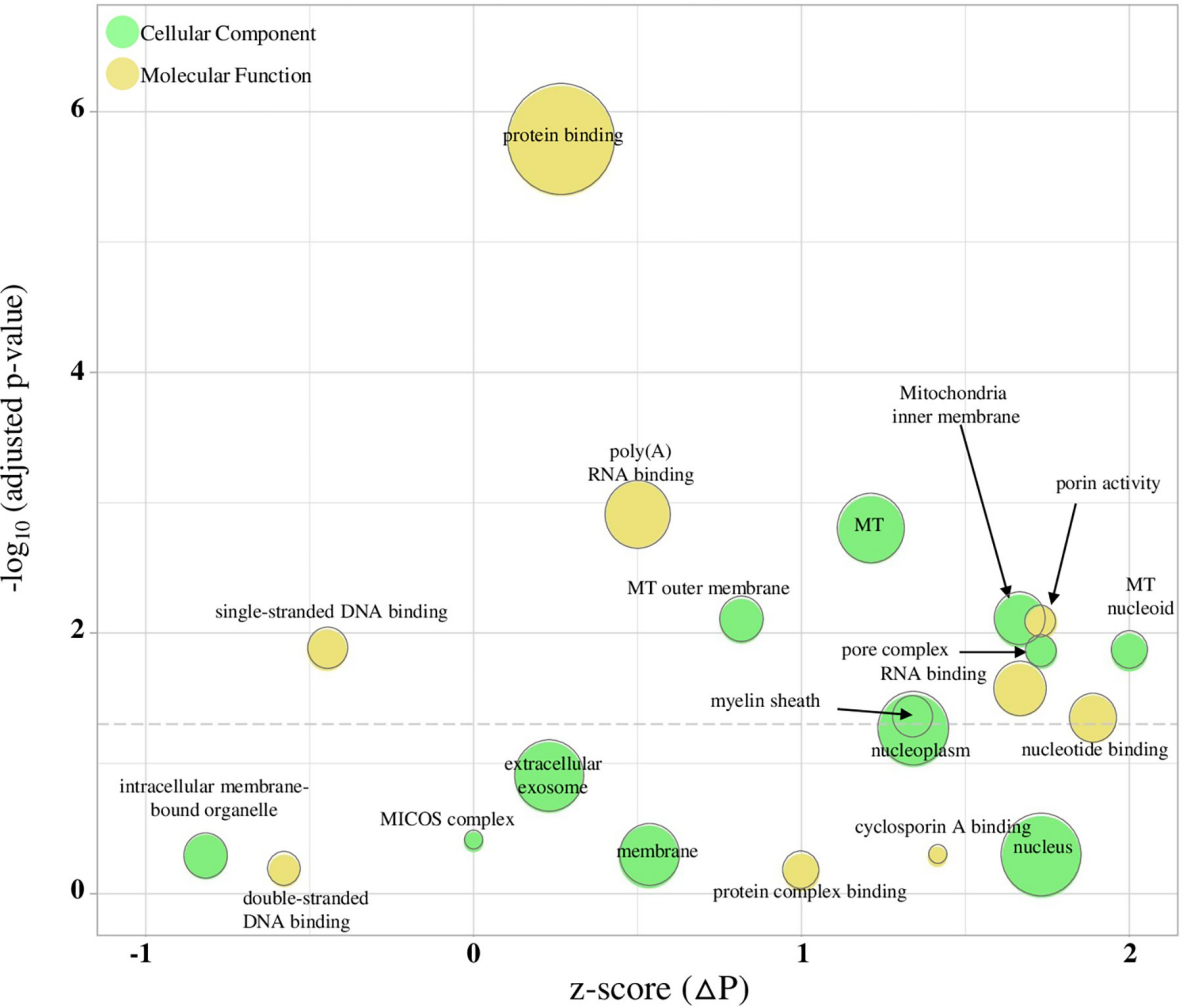
DAVID enrichment analysis of the ΔP proteome. ΔP was identified using the parameters indicated in Fig 1A plot of the DAVID enrichment analysis in which the bubble size describes the number of proteins within ΔP associated with each term. The x-axis is a GOPlot calculated z-score that indicates whether the term is likely increased (z > 0) or decreased (z < 0) based on the log_2_ abundance ratios of the proteins within the GO term. Terms that fall below the dotted grey line do not reach statistical significance as determined by an adjusted p-value ≥ 0.05 by Benjamini-Hochberg correction.

Based on the finding that Aβ_42_ can bind to the α7 nAChR via the orthosteric binding site [35], we tested the effect of receptor antagonism on the Aβ_42_ driven proteomic responses. We explored this by determining which proteins within Aβ_42_^P^ are returned to control baseline or are altered in an opposite direction by Aβ_42_ + Bgtx co-treatment. Proteins identified using this criterion are designated ΔP (Fig 1) and are listed in S4 Table in S1 File. DAVID enrichment was also used to analyze ΔP (December 2021). As shown in Fig 6, protein binding, mitochondria, and poly(A) RNA binding GO terms significantly overlap with ΔP, suggesting a role for α7nAChR in these amyloid related responses.

### Nicotinic receptors impact mitochondria and protein binding components of the proteome

**Fig 7:**
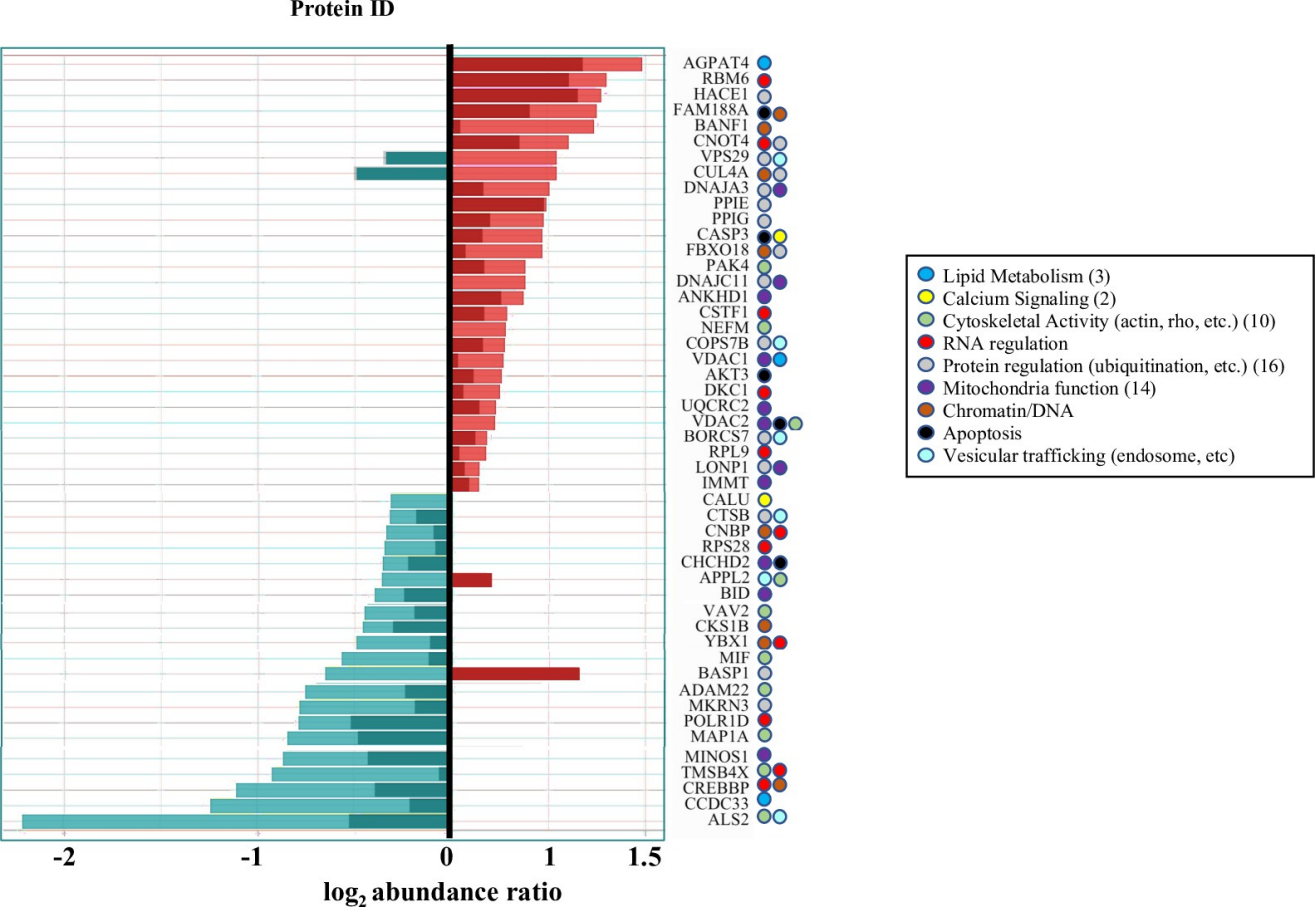
Protein binding components of ΔP. A change in protein levels measured by the log_2_ abundance ratio measures within the Aβ_42_ and Aβ_42_ + Bgtx treatment conditions. Color shading highlights the impact of treatment condition on protein expression. Light (Aβ_42_); dark (Aβ_42_ + Bgtx). The function of each protein is shown using colored dots.

DAVID analyses of ΔP suggests that “protein binding” relates Aβ_42_ and α7 nAChR proteome responses (Fig 7). Protein binding is a broad functional category however encompassing diverse proteins and processes that participate in interactions between not only proteins, but also proteins and other molecules including lipids and nucleic acids [36, 37]. A list of the proteins within the protein binding category from ΔP is presented in Fig 7. Quantitative changes are shown for each according to the treatment condition. A classification of each protein according to its function or subcellular localization based on UniProt is indicated. This classification shows the involvement of ΔP components in various cellular processes including mitochondria, cytoskeleton, and vesicular regulation. It is interesting to note that some proteins were found to be altered in the opposite direction by Aβ_42_ + Bgtx co-application relative to Aβ_42_ alone.

We found a subset of proteins that decreased in the presence of Aβ_42_ yet increased in the presence of Aβ_42_ + Bgtx. This includes: brain acidic soluble protein1 (BASP1), which regulates actin dynamics during axon growth [38, 39]; adaptor protein, phosphotyrosine interacting with PH domain and leucine zipper 2 (APPL2), an anchor proteins in endosomes [40]. Two other proteins were increased in the presence of Aβ_42_, but their expression was reduced in cells treated with Aβ_42_ + Bgtx: vacuolar protein sorting-associated protein 29 (VPS29) and cullin-4A (CUL4A). VPS29 is important in endo-lysosomal trafficking, a process impacted in both Parkinson’s and Alzheimer’s diseases [41]. CUL4A ubiquitin ligase activity regulates protein activity in favor of Aβ_42_ oligomerization [42].

**Fig 8:**
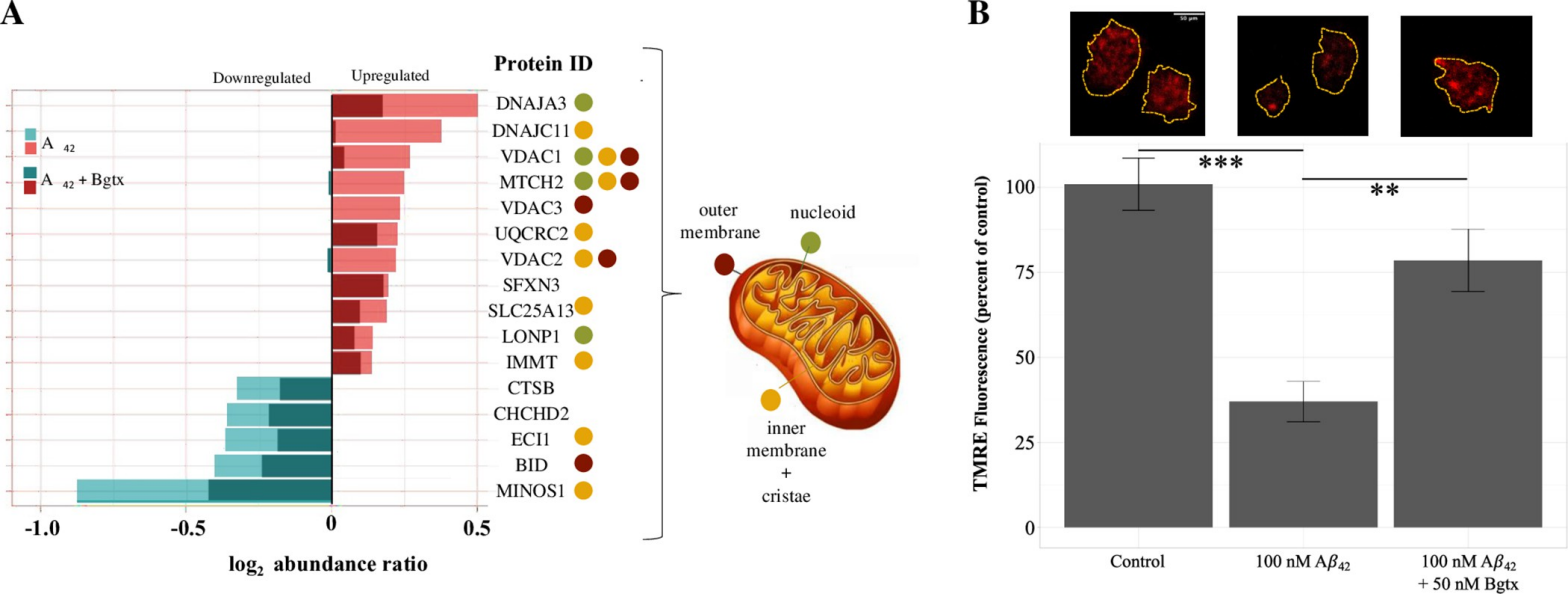
Mitochondrial proteins within ΔP. A) A change in protein level measured by the log_2_ abundance ratio measures within Aβ_42_ and Aβ_42_ + Bgtx treatment conditions. Color shading highlights the impact of treatment on protein expression. Light (Aβ_42_); dark (Aβ_42_ + Bgtx). The known localization of each protein within the mitochondria is shown using colored dots. B) Top, representative images for each condition showing fluorescence intensity of TMRE. Bottom, average percent change in TMRE fluorescence. (n ≤ 21). *p < 0.05; ** p < 0.01; p < 0.001.

Studies have shown that mitochondrial trafficking, metabolic activity, and calcium management are impacted during amyloid toxicity [43–45]. DAVID analysis of ΔP corroborates these findings and suggests the involvement of α7 nAChR in Aβ_42_ mitochondrial stress. The mitochondrial proteins within ΔP are shown in Fig 8A. The figure also indicates their mitochondrial location and quantitative change according to the treatment condition. Thus, our proteomic findings highlight the impact of Aβ_42_ treatment on mitochondria function through specifically altered proteins. To functionally test this, we used TMRE (cell permeant fluorescent dye used to measure mitochondrial membrane potential) to examine differences between cells treated for 72 hours with Aβ_42_ or Aβ_42_ + Bgtx and controls. The results show a significant reduction in TMRE fluorescence in the Aβ_42_ group (p = 8 x 10^−7^) but not in Aβ_42_ + Bgtx (p = 0.106) when compared to the controls (Fig 8B). In addition, we found a significant reduction in TMRE fluorescence between the Aβ_42_ and Aβ_42_ + Bgtx treatment groups (p = 0.001). Given that TMRE is a measure of mitochondrial membrane potential and bioenergetic function, these results suggest that Aβ_42_ exposure directly impacts mitochondrial activity in a manner that involves the α7 nAChR.

## Discussion

Amyloid plaques and tau aggregates are physical components of chronic neurodegeneration considered drivers of cognitive decline in AD [1]. It is widely accepted however, that cellular and synaptic dysfunction arise earlier than the emergence of clinical disease and/or the detection of amyloid plaques due to yet unknown processes that may involve amyloid protein processing and/or accumulation [46]. Paradoxically, Aβ_42_ containing plaques are seen in aging individuals without dementia, and also arise in response to brain trauma and stroke [47]. Thus, how amyloid processing leads to neurotoxicity in the course of AD remains unknown.

Soluble forms of the cleaved APP protein include the Aβ_42_ peptide, are known to have neurotoxic effects in cells as well as brain slices [48]. In fact, Aβ_42_ peptides alone can form highly stable oligomers (>50 kDa) that trigger tau disruptions [49]. It is well documented that Aβ_42_ promotes damage to neurons through oxidative stress, membrane ion permeability, and excitotoxicity. The identification of various cellular targets of Aβ_42_ including the α7 nAChR enables a framework for understanding and potentially treating amyloid toxicity in brain [10, 50, 51]. In one scenario, α7 nAChR binding has been shown to participate in the internalization and accumulation of Aβ_42_ in cholinergic neurons [48].

We utilized a proteomics approach to identify mechanisms of Aβ_42_ pathogenicity in the human neural SH-SY5Y cell line, which is a model for cholinergic degeneration and endogenously expresses *α*7 nAChRs that bind Aβ_42_ [11, 52, 53]. We compared the effects of Aβ_42_ treatment alone with Aβ_42_ co-applied with the selective α7 nAChR blocker, Bgtx, for 72 hours. This time course was chosen for an ability to examine proteome responses that can lead to neurotoxicity. The overall profile of proteins identified by MS in this study appear broadly comparable between the two treatment conditions (Aβ_42_ *vs*. Aβ_42_+Bgtx) as evidenced by their strong correlation. This observation suggests that Aβ_42_ is the dominant driver of proteomic change within these cells, and that it is possible that Aβ_42_-induced changes are mediated by several receptor pathways in these cells. Indeed SH-SY5Y cells express N-methyl-D-aspartate receptors (NMDAR), which are known to interact with Aβ_42_ [11]. It is noteworthy however that the cellular and molecular components of the response identified by bioinformatics points to proteomes enriched in differing GO terms. These differences underscore the actions of Aβ_42_ on pathways differentially impacted by Bgtx.

A bioinformatic analysis of the proteomic data shows that Aβ_42_^P^ is enriched in mitochondrial proteins confirming the involvement of mitochondria in Aβ_42_ pathogenicity [54]. Changes in specific mitochondrial proteins such as caspase 3 (CASP3) and BH3 interacting domain death agonist (BID) can contribute to apoptosis in the AD brain [55, 56]. In addition, CASP3 is known to cleave tau in a manner that encourages tangle formation consistent with findings that Aβ_42_ can trigger tau disruption in neurons [57]. Our proteomic results show that Bgtx co-application diminishes the effect of Aβ_42_ on several mitochondrial proteins including BID suggesting that α7 nAChRs are involved in amyloid associated mitochondrial dysfunction [58]. Experimental TMRE findings support this and confirm that α7 nAChR blockade with Bgtx significantly diminished the effect of Aβ_42_ on mitochondrial membrane depolarization.

Enrichment analysis of the Aβ_42_ + Bgtx co-treatment condition enabled the identification of the Aβ_42_/Bgtx^P^ proteome, which was different from Aβ_42_^P^. In particular, Aβ_42_/Bgtx^P^ is enriched in heterotrimeric G-protein components consistent with our earlier findings on nAChR signaling through various G-proteins [12, 59]. Proteomic evidence now suggests a role for G-protein activity in nAChR mediated Aβ_42_ responses [12, 13]. This is consistent with findings by Lasala, et al. that indicate that Aβ_42_ exposure can elicit direct conformational changes in the α7 nAChR which may thus affect its association with G-proteins in neural cells [60].

Along these lines, an enrichment analysis of the ΔP dataset was used to identify specific pathways that may functionally uncouple Aβ_42_ from α7 nAChR in SH-SY5Y cells. ΔP represents proteins that are returned to baseline (control) levels or are impacted in the opposite direction in response to Bgtx co-presentation. Analysis of ΔP using DAVID enrichment identifies changes in protein binding for a wide range of cellular targets. Protein complex formation (ETC Complex I), protein synthesis and degradation, and components of cytoskeletal activity are all noted within the ΔP protein network. APPL2 is an endosomal membrane anchor protein that participates in β-catenin and PI3K/Akt signaling pathways in neurite growth [40, 61] and chromatin remodeling [44]. Trafficking in the endo- and lysosomal pathways, which has been implicated in AD and other neurodegenerative disease [62], appears linked to ΔP components through several proteins including the retromer complex that includes vacuolar sorting protein 29 (VSP29). In our data, VSP29, a protein necessary for appropriate synaptic transmission [41], is found to be upregulated in the presence of Aβ_42_ but downregulated in the presence of Bgtx.

## Supporting information

S1 File (ZIP)
