# Supplementary material for: Nicotinic receptor components of amyloid beta 42 proteome regulation in human neural cells
Source: PLoS One. 2022 Aug 12;17(8):e0270479. doi: 10.1371/journal.pone.0270479 (PMC9374227; doi:10.1371/journal.pone.0270479)
Supplement: S1 File — (ZIP) [file pone.0270479.s001.zip › STable1Ab42.docx]

**Supplement Table 1: A𝛽_42_ Proteome (A𝛽_42_^P^)**

| **gene symbol** | **description** | **log_2_ fold change**  **A𝛽_42_** | **fold change A𝛽_42_**  **p-value** |
| --- | --- | --- | --- |
| RPS6KA5 | ribosomal protein S6 kinase alpha-5 isoform a | 2.18 X 10^+00^ | 5.25 X 10^-16^ |
| KRT1 | keratin, type II cytoskeletal 1 | -2.69 X 10^+00^ | 5.25 X 10^-16^ |
| ALS2 | alsin isoform X1 | -2.22 X 10^+00^ | 5.25 X 10^-16^ |
| BASP1 | brain acid soluble protein 1 | -6.57 X 10^-01^ | 2.00 X 10^-03^ |
| C9orf84 | uncharacterized protein C9orf84 isoform X1 | -9.05 X 10^-01^ | 2.48 X 10^-03^ |
| RBM41 | RNA-binding protein 41 isoform 4 | -8.52 X 10^-01^ | 1.21 X 10^-04^ |
| NME1 | nucleoside diphosphate kinase A isoform a | -6.99 X 10^-01^ | 3.86 X 10^-02^ |
| CCDC33 | coiled-coil domain-containing protein 33 isoform X1 | -1.25 X 10^+00^ | 4.89 X 10^-04^ |
| TMSB4X | thymosin beta-4 | -9.30 X 10^-01^ | 1.32 X 10^-04^ |
| MKRN3 | probable E3 ubiquitin-protein ligase makorin-3 | -7.86 X 10^-01^ | 8.22 X 10^-04^ |
| ADAM22 | disintegrin and metalloproteinase domain-containing protein 22 isoform X1 | -7.56 X 10^-01^ | 2.80 X 10^-02^ |
| APPL2 | DCC-interacting protein 13-beta isoform 2 | -3.66 X 10^-01^ | 1.01 X 10^-02^ |
| SUMO2 | small ubiquitin-related modifier 2 isoform a precursor | -3.20 X 10^-01^ | 4.89 X 10^-02^ |
| IL12RB2 | interleukin-12 receptor subunit beta-2 isoform X1 | -1.49 X 10^+00^ | 5.25 X 10^-16^ |
| BCAP31 | B-cell receptor-associated protein 31 isoform a | -5.40 X 10^-01^ | 3.23 X 10^-05^ |
| INTS12 | integrator complex subunit 12 | -2.32 X 10^+00^ | 5.25 X 10^-16^ |
| MINOS1 | MICOS complex subunit MIC10 isoform c | -8.73 X 10^-01^ | 8.82 X 10^-04^ |
| TMSB10 | thymosin beta-10 | -4.34 X 10^-01^ | 2.43 X 10^-04^ |
| PDCD10 | programmed cell death protein 10 isoform X1 | 5.39 X 10^-01^ | 6.92 X 10^-05^ |
| NUCKS1 | nuclear ubiquitous casein and cyclin-dependent kinase substrate 1 | -4.74 X 10^-01^ | 1.31 X 10^-05^ |
| CALU | calumenin isoform c precursor | -3.20 X 10^-01^ | 4.48 X 10^-02^ |
| CNBP | cellular nucleic acid-binding protein isoform 3 | -3.44 X 10^-01^ | 1.45 X 10^-02^ |
| POLR1D | DNA-directed RNA polymerases I and III subunit RPAC2 isoform 1 | -7.91 X 10^-01^ | 2.91 X 10^-02^ |
| ARID1A | AT-rich interactive domain-containing protein 1A isoform a | 6.07 X 10^-01^ | 1.51 X 10^-03^ |
| RPS28 | 40S ribosomal protein S28 | -3.51 X 10^-01^ | 2.80 X 10^-02^ |
| YBX1 | nuclease-sensitive element-binding protein 1 | -4.92 X 10^-01^ | 5.60 X 10^-06^ |
| VAV2 | guanine nucleotide exchange factor VAV2 isoform X1 | -4.52 X 10^-01^ | 2.80 X 10^-02^ |
| SNAP29 | synaptosomal-associated protein 29 | -1.19 X 10^+00^ | 3.67 X 10^-07^ |
| SMARCA4 | transcription activator BRG1 isoform A | 3.29 X 10^-01^ | 9.97 X 10^-07^ |
| BID | BH3-interacting domain death agonist isoform 1 | -4.02 X 10^-01^ | 2.80 X 10^-02^ |
| FAM118B | protein FAM118B isoform 1 | 9.25 X 10^-01^ | 1.55 X 10^-04^ |
| LGALS8 | galectin-8 isoform a | 1.04 X 10^+00^ | 7.96 X 10^-03^ |
| PCBD1 | pterin-4-alpha-carbinolamine dehydratase isoform 1 | 5.16 X 10-^01^ | 1.35 X 10^-06^ |
| NOM1 | nucleolar MIF4G domain-containing protein 1 isoform 2 | -1.78 X 10^+00^ | 3.06 X 10^-07^ |
| ATP5F1 | ATP synthase F(0) complex subunit B1, mitochondrial precursor | 2.80 X 10^-01^ | 1.41 X 10^-04^ |
| TRAPPC2L | trafficking protein particle complex subunit 2-like protein isoform 1 | 3.98 X 10^-01^ | 4.82 X 10^-03^ |
| CLTA | clathrin light chain A isoform a | -4.86 X 10^-01^ | 1.46 X 10^-05^ |
| AK6 | adenylate kinase isoenzyme 6 isoform b | 3.98 X 10^-01^ | 2.09 X 10-02 |
| PPP1CC | serine/threonine-protein phosphatase PP1-gamma catalytic subunit isoform X1 | 9.45 X 10^-01^ | 1.48 X 10^-08^ |
| CTSB | cathepsin B isoform X1 | -3.24 X 10^-01^ | 3.54 X 10^-02^ |
| CKS1B | cyclin-dependent kinases regulatory subunit 1 | -4.60 X 10^-01^ | 1.34 X 10^-03^ |
| SDHD | succinate dehydrogenase [ubiquinone] cytochrome b small subunit, mitochondrial isoform a precursor | 7.68 X 10^-01^ | 1.46 X 10^-02^ |
| TMEM161A | transmembrane protein 161A isoform 1 precursor | -1.13 X 10^+00^ | 1.50 X 10^-10^ |
| MYO1B | unconventional myosin-Ib isoform 1 | 2.23 X 10^-01^ | 5.94 X 10^-04^ |
| MAP1A | microtubule-associated protein 1A | -8.49 X 10^-01^ | 1.98 X 10^-02^ |
| HMG20B | SWI/SNF-related matrix-associated actin-dependent regulator of chromatin subfamily E member 1-related | -6.99 X 10^-01^ | 2.91 X 10^-06^ |
| NUMBL | numb-like protein isoform a | -2.91 X 10^+00^ | 5.25 X 10^-16^ |
| TBR1 | T-box brain protein 1 | -9.32 X 10^-01^ | 1.98 X 10^-08^ |
| SLC25A11 | mitochondrial 2-oxoglutarate/malate carrier protein isoform 1 | 3.95 X 10^-01^ | 1.41 X 10^-04^ |
| CHCHD2 | coiled-coil-helix-coiled-coil-helix domain-containing protein 2 precursor isoform 1 | -3.60 X 10^-01^ | 2.23 X 10^-02^ |
| ENO3 | beta-enolase isoform X1 | 1.58 X 10^-01^ | 1.41 X 10^-02^ |
| RPS15 | 40S ribosomal protein S15 isoform 1 | -4.82 X 10^-01^ | 6.30 X 10^-06^ |
| ATP5L | ATP synthase subunit g, mitochondrial | 1.56 X 10^-01^ | 1.82 X 10^-02^ |
| PPIG | peptidyl-prolyl cis-trans isomerase G | 4.75 X 10^-01^ | 1.88 X 10^-02^ |
| COA4 | cytochrome c oxidase assembly factor 4 homolog, mitochondrial isoform X1 | 1.78 X 10^+00^ | 5.25 X 10^-16^ |
| MIF | macrophage migration inhibitory factor | -5.73 X 10^-01^ | 1.14 X 10^-08^ |
| RAB6A | ras-related protein Rab-6A isoform a | 1.95 X 10^-01^ | 1.12 X 10^-02^ |
| AKT3 | RAC-gamma serine/threonine-protein kinase isoform X1 | 2.58 X 10^-01^ | 2.44 X 10^-02^ |
| SATB1 | DNA-binding protein SATB1 isoform 2 | 4.19 X 10^-01^ | 2.52 X 10^-02^ |
| ZNF181 | zinc finger protein 181 isoform X1 | 3.22 X 10^-01^ | 3.62 X 10^-03^ |
| GGH | gamma-glutamyl hydrolase precursor | 1.81 X 10^-01^ | 4.52 X 10^-02^ |
| CUTA | protein CutA isoform 1 | 2.04 X 10^-01^ | 2.91 X 10^-02^ |
| PPIE | peptidyl-prolyl cis-trans isomerase E isoform 4 | 4.89 X 10^-01^ | 2.50 X 10^-02^ |
| CDC14B | dual specificity protein phosphatase CDC14B isoform X9 | 5.56 X 10^-01^ | 2.94 X 10^-05^ |
| FAHD1 | acylpyruvase FAHD1, mitochondrial isoform 1 | 2.77 X 10^-01^ | 1.57 X 10^-02^ |
| ISYNA1 | inositol-3-phosphate synthase 1 isoform X3 | 2.61 X 10^-01^ | 2.08 X 10^-04^ |
| NEDD8-MDP1 | NEDD8-MDP1 protein | -3.33 X 10^-01^ | 4.21 X 10^-02^ |
| ECI1 | enoyl-CoA delta isomerase 1, mitochondrial isoform 1 precursor | -3.64 X 10^-01^ | 6.93 X 10^-03^ |
| KRT10 | keratin, type I cytoskeletal 10 isoform X1 | -1.19 X 10^+00^ | 5.25 X 10^-16^ |
| SFXN3 | sideroflexin-3 | 1.95 X 10^-01^ | 9.49 X 10^-03^ |
| UQCRC2 | cytochrome b-c1 complex subunit 2, mitochondrial precursor | 2.28 X 10^-01^ | 1.14 X 10^-02^ |
| C17orf49 | chromatin complexes subunit BAP18 isoform 1 | 5.25 X 10^-01^ | 4.21 X 10^-02^ |
| LONP1 | lon protease homolog, mitochondrial isoform 1 precursor | 1.43 X 10^-01^ | 2.92 X 10^-02^ |
| BORCS7 | BLOC-1-related complex subunit 7 | 1.81 X 10^-01^ | 2.56 X 10^-02^ |
| RNLS | renalase isoform 1 precursor | 2.82 X 10^-01^ | 1.02 X 10^-02^ |
| MGST3 | microsomal glutathione S-transferase 3 isoform X1 | 1.65 X 10^-01^ | 3.79 X 10^-02^ |
| THEM6 | protein THEM6 precursor | 2.40 X 10^-01^ | 2.24 X 10^-04^ |
| GGT7 | glutathione hydrolase 7 isoform 1 | -5.51 X 10^+00^ | 5.25 X 10^-16^ |
| IMMT | MICOS complex subunit MIC60 isoform X1 | 1.40 X 10^-01^ | 4.21 X 10^-02^ |
| SLC25A13 | calcium-binding mitochondrial carrier protein Aralar2 isoform X1 | 1.90 X 10^-01^ | 4.63 X 10^-03^ |
| KRT2 | keratin, type II cytoskeletal 2 epidermal | -1.25 X 10^+00^ | 5.25 X 10^-16^ |
| ANKHD1-EIF4EBP3 | ANKHD1-EIF4EBP3 protein | 3.71 X 10^-01^ | 3.12 X 10^-02^ |
| COPS7B | COP9 signalosome complex subunit 7b isoform b | 2.74 X 10^-01^ | 2.73 X 10^-03^ |
| PPP2R2D | serine/threonine-protein phosphatase 2A 55 kDa regulatory subunit B delta isoform isoform a | 1.06 X 10^+00^ | 1.36 X 10^-08^ |
| HACE1 | E3 ubiquitin-protein ligase HACE1 isoform a | 7.71 X 10^-01^ | 1.09 X 10^-02^ |
| DNAJA3 | dnaJ homolog subfamily A member 3, mitochondrial isoform 1 | 5.03 X 10^-01^ | 2.46 X 10^-04^ |
| BCL7C | B-cell CLL/lymphoma 7 protein family member C isoform X1 | -5.21 X 10^-01^ | 8.73 X 10^-03^ |
| TRMT2A | tRNA (uracil-5-)-methyltransferase homolog A isoform c | 4.59 X 10^-01^ | 2.55 X 10^-02^ |
| TMEM106B | transmembrane protein 106B | -5.46 X 10^-01^ | 2.86 X 10^-02^ |
| RABGEF1 | rab5 GDP/GTP exchange factor isoform b | 1.11 X 10^+00^ | 4.02 X 10^-04^ |
| BCAT2 | branched-chain-amino-acid aminotransferase, mitochondrial isoform a precursor | 9.78 X 10^-01^ | 7.50 X 10^-03^ |
| SSSCA1 | Sjoegren syndrome/scleroderma autoantigen 1 isoform 1 | -6.10 X 10^-01^ | 7.96 X 10^-03^ |
| TDP2 | tyrosyl-DNA phosphodiesterase 2 | -8.06 X 10^-01^ | 9.13 X 10^-03^ |
| LONP2 | lon protease homolog 2, peroxisomal isoform 3 | 9.71 X 10^-01^ | 4.07 X 10^-05^ |
| CREBBP | CREB-binding protein isoform a | -1.11 X 10^+00^ | 4.78 X 10^-10^ |
| CCDC66 | coiled-coil domain-containing protein 66 isoform 3 | -6.15 X 10-01 | 2.50 X 10-02 |
| CDIPT | CDP-diacylglycerol--inositol 3-phosphatidyltransferase isoform X1 | 1.30 X 10^+00^ | 2.17 X 10^-06^ |
| PAK4 | serine/threonine-protein kinase PAK 4 isoform X1 | 3.81 X 10^-01^ | 2.61 X 10^-03^ |
| SCOC | short coiled-coil protein isoform 1 | -6.28 X 10^-01^ | 2.53 X 10^-04^ |
| FAM186B | protein FAM186B isoform X1 | -5.33 X 10^-01^ | 2.48 X 10^-04^ |
| MRPS10 | 28S ribosomal protein S10, mitochondrial isoform X1 | 6.31 X 10^-01^ | 5.64 X 10^-04^ |
| RPL9 | 60S ribosomal protein L9 | 1.76 X 10^-01^ | 6.85 X 10^-03^ |
| CASP3 | caspase-3 isoform a preproprotein | 4.68 X 10-01 | 5.47 X 10^-08^ |
| DKC1 | H/ACA ribonucleoprotein complex subunit 4 isoform 1 | 2.49 X 10^-01^ | 4.11 X 10^-02^ |
| SRGAP1 | SLIT-ROBO Rho GTPase-activating protein 1 isoform 1 | 9.17 X 10^-01^ | 1.91 X 10^-05^ |
| CSTF1 | cleavage stimulation factor subunit 1 | 2.86 X 10^-01^ | 1.46 X 10^-02^ |
| HMGN2 | non-histone chromosomal protein HMG-17 | 6.08 X 10^-01^ | 2.36 X 10^-04^ |
| RBM6 | RNA-binding protein 6 isoform 1 | 7.98 X 10^-01^ | 1.21 X 10^-02^ |
| COA3 | cytochrome c oxidase assembly factor 3 homolog, mitochondrial | -6.85 X 10^-01^ | 4.13 X 10^-03^ |
| DIP2B | disco-interacting protein 2 homolog B | 2.93 X 10^-01^ | 2.38 X 10^-02^ |
| MTCH2 | mitochondrial carrier homolog 2 isoform 1x | 2.50 X 10^-01^ | 4.35 X 10^-03^ |
| VDAC1 | voltage-dependent anion-selective channel protein 1 isoform X1 | 2.68 X 10^-01^ | 4.40 X 10^-05^ |
| BAG1 | BAG family molecular chaperone regulator 1 isoform BAG-1L | 8.16 X 10^-01^ | 5.64 X 10^-04^ |
| VDAC3 | voltage-dependent anion-selective channel protein 3 isoform 2 | 2.36 X 10^-01^ | 3.45 X 10^-04^ |
| DNAJC11 | dnaJ homolog subfamily C member 11 | 3.79 X 10^-01^ | 7.96 X 10^-03^ |
| CNOT4 | CCR4-NOT transcription complex subunit 4 isoform f | 6.03 X 10^-01^ | 2.79 X 10^-02^ |
| MED23 | mediator of RNA polymerase II transcription subunit 23 isoform X1 | -7.71 X 10^-01^ | 4.64 X 10^-02^ |
| HEATR5A | HEAT repeat-containing protein 5A | 1.42 X 10^+00^ | 1.55 X 10^-12^ |
| VDAC2 | voltage-dependent anion-selective channel protein 2 isoform 1 | 2.23 X 10^-01^ | 5.88 X 10^-04^ |
| NEFM | neurofilament medium polypeptide isoform 1 | 2.79 X 10^-01^ | 2.63 X 10^-02^ |
| EPB41L4A | band 4.1-like protein 4A isoform 1 | 5.15 X 10^-01^ | 3.78 X 10^-03^ |
| MAGI3 | membrane-associated guanylate kinase, WW and PDZ domain-containing protein 3 isoform 1 | -1.23 X 10^+00^ | 5.27 X 10^-09^ |
| AGPAT4 | 1-acyl-sn-glycerol-3-phosphate acyltransferase delta | 9.83 X 10^-01^ | 9.57 X 10^-04^ |
| DPM3 | dolichol-phosphate mannosyltransferase subunit 3 isoform 1 | 1.33 X 10^+00^ | 5.25 X 10-^16^ |
| FAM188A | ubiquitin carboxyl-terminal hydrolase MINDY-3 isoform a | 7.47 X 10^-01^ | 1.55 X 10^-04^ |
| FAM45A | protein FAM45A isoform 1 | 1.12 X 10^+00^ | 2.90 X 10^-07^ |
| UMODL1 | uromodulin-like 1 isoform 2 precursor | 1.07 X 10^+00^ | 6.30 X 10^-12^ |
| CCNK | cyclin-K | -7.54 X 10^-01^ | 5.00 X 10^-02^ |
| FBXO18 | F-box DNA helicase 1 isoform X1 | 4.67 X 10^-01^ | 1.83 X 10^-03^ |
| TANGO6 | transport and Golgi organization protein 6 homolog | 9.40 X 10-^01^ | 8.22 X 10^-03^ |
| LARP4 | la-related protein 4 isoform X1 | -1.04 X 10^+00^ | 1.17 X 10^-04^ |
| VPS29 | vacuolar protein sorting-associated protein 29 isoform 1 | 5.41 X 10^-01^ | 7.22 X 10^-04^ |
| BANF1 | barrier-to-autointegration factor isoform X1 | 7.33 X 10^-01^ | 1.28 X 10^-09^ |
| CCDC169-SOHLH2 | CCDC169-SOHLH2 protein | -2.98 X 10^+00^ | 5.25 X 10^-16^ |
| HAUS8 | HAUS augmin-like complex subunit 8 isoform a | 6.62 X 10^-01^ | 1.18 X 10^-03^ |
| TUBB8 | tubulin beta-8 chain | -2.26 X 10^+00^ | 5.25 X 10^-16^ |
| CUL4A | cullin-4A isoform 1 | 5.41 X 10^-01^ | 2.80 X 10-^02^ |
