# Supplementary material for: Nicotinic receptor components of amyloid beta 42 proteome regulation in human neural cells
Source: PLoS One. 2022 Aug 12;17(8):e0270479. doi: 10.1371/journal.pone.0270479 (PMC9374227; doi:10.1371/journal.pone.0270479)
Supplement: S1 File — (ZIP) [file pone.0270479.s001.zip › STable2AbrevP.docx]

**Supplement Table 2: A𝛽_rev_ Protein Changes**

| **gene symbol** | **description** | **log_2_ fold change**  **A𝛽_rev_** | **fold change A𝛽_rev_**  **p-value** |
| --- | --- | --- | --- |
| PFDN4 | prefoldin subunit 4 | 3.12 X 10^+00^ | 6.46 X 10^-06^ |
|  | vesicle transport through interaction with t-SNAREs homolog 1A isoform X1 | 2.76 X 10^+00^ | 7.11 X 10^-03^ |
| CEBPB | CCAAT/enhancer-binding protein beta isoform a | 2.72 X 10^+00^ | 5.27 X 10^-05^ |
| RSBN1L | round spermatid basic protein 1-like protein | 2.38 X 10^+00^ | 2.52 X 10^-02^ |
| NDUFA12 | NADH dehydrogenase [ubiquinone] 1 alpha subcomplex subunit 12 isoform a | 2.33 X 10^+00^ | 2.89 X 10^-02^ |
| PPIL2 | peptidyl-prolyl cis-trans isomerase-like 2 isoform X1 | 2.04 X 10^+00^ | 2.56 X 10^-02^ |
| RIDA | 2-iminobutanoate/2-iminopropanoate deaminase | 1.98 X 10^+00^ | 3.25 X 10^-02^ |
| PLAU | urokinase-type plasminogen activator isoform 1 preproprotein | 1.93 X 10^+00^ | 2.22 X 10-^02^ |
| ATP13A2 | cation-transporting ATPase 13A2 isoform X1 | 1.91 X 10^+00^ | 6.67 X 10^-03^ |
| HBA1 | hemoglobin subunit alpha | 1.76 X 10^+00^ | 4.25 X 10^-03^ |
| MIF | macrophage migration inhibitory factor | 1.74 X 10^+00^ | 3.19 X 10-^04^ |
| CUTA | protein CutA isoform 1 | 1.68 X 10^+00^ | 6.45 X 10^-04^ |
| TUBB1 | tubulin beta-1 chain | 1.63 X 10^+00^ | 1.45 X 10^-02^ |
| PHPT1 | 14 kDa phosphohistidine phosphatase isoform 3 | 1.47 X 10^+00^ | 5.52 X 10^-03^ |
| NUB1 | NEDD8 ultimate buster 1 isoform 1 | 1.31 X 10^+00^ | 8.11 X 10-^03^ |
| EPCAM | epithelial cell adhesion molecule precursor | 1.29 X 10^+00^ | 1.50 X 10^-02^ |
| CPLX2 | complexin-2 | 1.17 X 10^+00^ | 2.68 X 10^-02^ |
| POTEE | POTE ankyrin domain family member E isoform X3 | 1.15 X 10^+00^ | 3.06 X 10^-02^ |
| AHNAK | neuroblast differentiation-associated protein AHNAK isoform X1 | 1.01 X 10^+00^ | 4.97 X 10^-02^ |
| CEP170 | centrosomal protein of 170 kDa isoform X1 | -1.61 X 10^+00^ | 3.32 X 10^-02^ |
| H1-5 | histone H1.5 | -1.68 X 10^+00^ | 4.83 X 10^-02^ |
| RPL18A | 60S ribosomal protein L18a | -1.69 X 10^+00^ | 4.61 X 10^-02^ |
| RPS16 | 40S ribosomal protein S16 isoform 1 | -1.72 X 10^+00^ | 3.98 X 10^-02^ |
| TBCB | tubulin-folding cofactor B isoform 1 | -1.72 X 10^+00^ | 3.81 X 10^-02^ |
| CPSF7 | cleavage and polyadenylation specificity factor subunit 7 isoform 1 | -1.72 X 10^+00^ | 4.95 X 10^-02^ |
| SNRPD1 | small nuclear ribonucleoprotein Sm D1 isoform 1 | -1.75 X 10^+00^ | 2.92 X 10^-02^ |
| RPL18 | 60S ribosomal protein L18 isoform 1 | -1.76 X 10^+00^ | 3.18 X 10^-02^ |
| HNRNPA2B1 | heterogeneous nuclear ribonucleoproteins A2/B1 isoform X1 | -1.78 X 10^+00^ | 2.82 X 10^-02^ |
| CSE1L | exportin-2 isoform 1 | -1.80 X 10^+00^ | 2.05 X 10^-02^ |
| RPS29 | 40S ribosomal protein S29 isoform 1 | -1.81 X 10^+00^ | 1.50 X 10^-02^ |
| ARHGDIA | rho GDP-dissociation inhibitor 1 isoform a | -1.82 X 10^+00^ | 2.17 X 10^-02^ |
| ACOT9 | acyl-coenzyme A thioesterase 9, mitochondrial isoform b precursor | -1.84 X 10^+00^ | 4.73 X 10^-02^ |
| PRKACA | cAMP-dependent protein kinase catalytic subunit alpha isoform 3 | -1.85 X 10^+00^ | 4.49 X 10^-02^ |
| SRSF3 | serine/arginine-rich splicing factor 3 | -1.85 X 10^+00^ | 1.81 X 10^-02^ |
| CALU | calumenin isoform c precursor | -1.86 X 10^+00^ | 7.11 X 10^-03^ |
|  | U6 snRNA-associated Sm-like protein LSm7 isoform X1 | -1.86 X 10^+00^ | 1.97 X 10^-02^ |
| PSMB5 | proteasome subunit beta type-5 isoform 1 | -1.86 X 10^+00^ | 1.38 X 10^-02^ |
|  | serine/threonine-protein phosphatase 5 isoform X1 | -1.88 X 10^+00^ | 2.94 X 10^-02^ |
| LAMTOR5 | ragulator complex protein LAMTOR5 | -1.89 X 10^+00^ | 3.08 X 10^-02^ |
| RDX | radixin isoform 1 | -1.90 X 10^+00^ | 1.13 X 10^-02^ |
| PSMB6 | proteasome subunit beta type-6 isoform 1 precursor | -1.92 X 10^+00^ | 6.33 X 10^-03^ |
| RPL31 | 60S ribosomal protein L31 isoform 1 | -1.94 X 10^+00^ | 6.80 X 10^-03^ |
| RPL15 | 60S ribosomal protein L15 isoform 1 | -1.95 X 10^+00^ | 9.93 X 10^-03^ |
| H1-3 | histone H1.3 | -1.98 X 10^+00^ | 7.74 X 10^-03^ |
| RPS20 | 40S ribosomal protein S20 isoform 1 | -1.98 X 10^+00^ | 7.75 X 10^-03^ |
| ACTR10 | actin-related protein 10 isoform X1 | -2.01 X 10^+00^ | 2.37 X 10^-02^ |
| AMT | aminomethyltransferase, mitochondrial isoform 1 precursor | -2.01 X 10^+00^ | 3.29 X 10^-02^ |
| SFRS2 | serine/arginine-rich splicing factor 2 isoform X1 | -2.01 X 10^+00^ | 1.02 X 10^-02^ |
| PPM1G | protein phosphatase 1G | -2.02 X 10^+00^ | 6.00 X 10^-03^ |
| POLA1 | DNA polymerase alpha catalytic subunit isoform 1 | -2.03 X 10^+00^ | 4.60 X 10^-02^ |
| CALD1 | caldesmon isoform 4 | -2.03 X 10^+00^ | 1.97 X 10^-02^ |
| SNRPF | small nuclear ribonucleoprotein F | -2.03 X 10^+00^ | 1.24 X 10^-02^ |
|  | nicotinate-nucleotide pyrophosphorylase [carboxylating] isoform X1 | -2.05 X 10^+00^ | 1.47 X 10^-02^ |
| CAPNS1 | calpain small subunit 1 isoform X1 | -2.06 X 10^+00^ | 1.92 X 10^-02^ |
|  | zinc finger protein 579 isoform X1 | -2.10 X 10^+00^ | 2.67 X 10^-02^ |
| EIF4H | eukaryotic translation initiation factor 4H isoform 1 | -2.15 X 10^+00^ | 2.63 X 10^-03^ |
| RPL28 | 60S ribosomal protein L28 isoform X1 | -2.15 X 10^+00^ | 2.59 X 10^-03^ |
| RPL34 | 60S ribosomal protein L34 | -2.15 X 10^+00^ | 2.54 X 10^-03^ |
| TBCA | tubulin-specific chaperone A isoform 2 | -2.18 X 10^+00^ | 1.97 X 10^-03^ |
| GATAD2B | transcriptional repressor p66-beta | -2.18 X 10^+00^ | 4.87 X 10^-02^ |
|  | NLR family CARD domain-containing protein 3 isoform X1 | -2.18 X 10^+00^ | 4.87 X 10^-02^ |
| HMGB2 | high mobility group protein B2 | -2.20 X 10^+00^ | 1.77 X 10^-03^ |
|  | general transcription factor 3C polypeptide 2 isoform X1 | -2.20 X 10^+00^ | 2.35 X 10^-02^ |
| ACAD9 | acyl-CoA dehydrogenase family member 9, mitochondrial | -2.22 X 10^+00^ | 1.40 X 10^-02^ |
| AMPD2 | AMP deaminase 2 isoform 1 | -2.23 X 10^+00^ | 1.89 X 10^-02^ |
| GNAI3 | guanine nucleotide-binding protein G(k) subunit alpha | -2.23 X 10^+00^ | 1.02 X 10^-02^ |
| DDC | aromatic-L-amino-acid decarboxylase isoform 1 | -2.24 X 10^+00^ | 1.35 X 10^-02^ |
| ME1 | NADP-dependent malic enzyme | -2.25 X 10^+00^ | 1.45 X 10^-02^ |
| YWHAQ | 14-3-3 protein theta | -2.26 X 10^+00^ | 1.08 X 10^-03^ |
| NSFL1C | NSFL1 cofactor p47 isoform X1 | -2.30 X 10^+00^ | 7.66 X 10^-03^ |
| SPCS2 | signal peptidase complex subunit 2 | -2.31 X 10^+00^ | 3.17 X 10^-03^ |
| CHCHD3 | MICOS complex subunit MIC19 isoform 1 | -2.34 X 10^+00^ | 3.67 X 10^-03^ |
| DHCR7 | 7-dehydrocholesterol reductase | -2.38 X 10^+00^ | 7.22 X 10^-03^ |
| STXBP1 | syntaxin-binding protein 1 isoform a | -2.39 X 10^+00^ | 1.84 X 10^-04^ |
| DCTN3 | dynactin subunit 3 isoform 1 | -2.39 X 10^+00^ | 4.53 X 10^-03^ |
| TRIR | telomerase RNA component interacting RNase isoform 1 | -2.40 X 10^+00^ | 9.84 X 10^-03^ |
|  | AP2-associated protein kinase 1 | -2.41 X 10^+00^ | 1.78 X 10^-02^ |
| MAP4 | microtubule-associated protein 4 isoform X1 | -2.41 X 10^+00^ | 1.41 X 10^-03^ |
| SBDS | ribosome maturation protein SBDS | -2.44 X 10^+00^ | 1.14 X 10^-03^ |
| RPL37A | 60S ribosomal protein L37a | -2.44 X 10^+00^ | 6.68 X 10^-05^ |
| TIMM10 | mitochondrial import inner membrane translocase subunit Tim10 isoform X1 | -2.45 X 10^+00^ | 2.07 X 10^-02^ |
| ZNF706 | zinc finger protein 706 isoform X1 | -2.46 X 10^+00^ | 1.37 X 10^-02^ |
| NUCKS1 | nuclear ubiquitous casein and cyclin-dependent kinase substrate 1 | -2.47 X 10^+00^ | 1.37 X 10-^03^ |
| HACD | very-long-chain (3R)-3-hydroxyacyl-CoA dehydratase 3 | -2.50 X 10^+00^ | 1.50 X 10^-03^ |
| SAFB | scaffold attachment factor B1 isoform 1 | -2.53 X 10^+00^ | 5.60 X 10^-03^ |
| FKBP9 | peptidyl-prolyl cis-trans isomerase FKBP9 isoform 1 precursor | -2.55 X 10^+00^ | 3.78 X 10^-03^ |
| CTTN | src substrate cortactin isoform a | -2.57 X 10^+00^ | 2.43 X 10^-04^ |
| CHMP1A | charged multivesicular body protein 1a isoform 2 | -2.57 X 10^+00^ | 2.62 X 10^-03^ |
| NDUFA7 | NADH dehydrogenase [ubiquinone] 1 alpha subcomplex subunit 7 | -2.57 X 10^+00^ | 3.26 X 10^-03^ |
| RPS27 | 40S ribosomal protein S27 isoform 1 | -2.59 X 10^+00^ | 1.42 X 10^-04^ |
| ATP5F1D | ATP synthase subunit delta, mitochondrial precursor | -2.60 X 10^+00^ | 2.70 X 10^-03^ |
| COPS3 | COP9 signalosome complex subunit 3 isoform 1 | -2.63 X 10^+00^ | 1.05 X 10^-03^ |
| BMP7 | bone morphogenetic protein 7 preproprotein | -2.63 X 10^+00^ | 3.34 X 10^-02^ |
| PAFAH1B1 | platelet-activating factor acetylhydrolase IB subunit alpha isoform X1 | -2.63 X 10^+00^ | 3.26 X 10^-04^ |
| CLTA | clathrin light chain A isoform a | -2.65 X 10^+00^ | 9.53 X 10^-06^ |
| TPM3 | tropomyosin alpha-3 chain isoform Tpm3.1cy | -2.67 X 10^+00^ | 2.68 X 10^-05^ |
| THOC3 | THO complex subunit 3 | -2.67 X 10^+00^ | 2.40 X 10^-03^ |
| CLPTM1 | cleft lip and palate transmembrane protein 1 isoform 2 | -2.68 X 10^+00^ | 2.94 X 10^-02^ |
| RAB25 | ras-related protein Rab-11B | -2.70 X 10^+00^ | 6.45 X 10^-06^ |
| SUMO2 | small ubiquitin-related modifier 2 isoform a precursor | -2.72 X 10^+00^ | 9.99 X 10^-06^ |
| SLC16A1 | monocarboxylate transporter 1 | -2.73 X 10^+00^ | 4.87 X 10^-02^ |
| NAPB | beta-soluble NSF attachment protein isoform b | -2.74 X 10^+00^ | 2.19 X 10^-02^ |
| PCYOX1 | prenylcysteine oxidase 1 precursor | -2.85 X 10^+00^ | 1.74 X 10^-02^ |
| NF1 | neurofibromin isoform 1 | -2.86 X 10^+00^ | 2.92 X 10-^02^ |
| SEC62 | translocation protein SEC62 | -2.87 X 10^+00^ | 6.48 X 10^-04^ |
| TLK2 | serine/threonine-protein kinase tousled-like 2 isoform X3 | -2.88 X 10^+00^ | 2.68 X 10^-02^ |
| GM2A | ganglioside GM2 activator isoform 1 precursor | -2.89 X 10^+00^ | 3.03 X 10^-03^ |
| CDK5RAP3 | CDK5 regulatory subunit-associated protein 3 isoform c | -2.90 X 10^+00^ | 3.18 X 10^-02^ |
| SERBP1 | plasminogen activator inhibitor 1 RNA-binding protein isoform 1 | -2.91 X 10^+00^ | 3.17 X 10^-06^ |
| PTMS | parathymosin isoform 2 | -2.93 X 10^+00^ | 5.51 X 10^-06^ |
| BABAM1 | BRISC and BRCA1-A complex member 1 isoform 1 | -2.95 X 10^+00^ | 3.85 X 10^-03^ |
| LSM6 | U6 snRNA-associated Sm-like protein LSm6 isoform X1 | -2.99 X 10^+00^ | 5.48 X 10^-06^ |
| NOL4L | nucleolar protein 4-like isoform 1 | -3.00 X 10^+00^ | 4.19 X 10^-03^ |
| RPL36AL | 60S ribosomal protein L36a-like | -3.01 X 10^+00^ | 5.73 X 10^-07^ |
| DIABLO | diablo homolog, mitochondrial isoform 1 precursor | -3.02 X 10^+00^ | 2.09 X 10^-05^ |
| EIF4B | eukaryotic translation initiation factor 4B isoform 1 | -3.02 X 10^+00^ | 1.88 X 10^-05^ |
| NME7 | nucleoside diphosphate kinase 7 isoform a | -3.04 X 10^+00^ | 1.47 X 10^-04^ |
| GHDC | GH3 domain-containing protein isoform 1 precursor | -3.04 X 10^+00^ | 2.96 X 10^-05^ |
| EDF1 | endothelial differentiation-related factor 1 isoform alpha | -3.05 X 10^+00^ | 2.27 X 10^-05^ |
| HMGN1 | non-histone chromosomal protein HMG-14 | -3.10 X 10^+00^ | 4.95 X 10^-02^ |
| ATP5IF1 | ATPase inhibitor, mitochondrial isoform 1 precursor | -3.15 X 10^+00^ | 6.03 X 10^-05^ |
| TBC1D10B | TBC1 domain family member 10B | -3.15 X 10^+00^ | 2.81 X 10^-02^ |
| CHMP2A | charged multivesicular body protein 2a isoform X1 | -3.18 X 10^+00^ | 4.03 X 10^-06^ |
| ATP5F1E | ATP synthase subunit epsilon, mitochondrial | -3.20 X 10^+00^ | 1.17 X 10^-05^ |
| TLR7 | toll-like receptor 7 precursor | -3.21 X 10^+00^ | 4.74 X 10^-05^ |
|  | zinc finger protein 638 isoform X1 | -3.22 X 10^+00^ | 3.39 X 10^-02^ |
| NME1 | nucleoside diphosphate kinase A isoform a | -3.27 X 10^+00^ | 1.75 X 10^-06^ |
| MRPS12 | 28S ribosomal protein S12, mitochondrial precursor | -3.28 X 10^+00^ | 2.09 X 10^-03^ |
| RPL37 | 60S ribosomal protein L37 | -3.31 X 10^+00^ | 4.95 X 10^-06^ |
| KHDRBS1 | KH domain-containing, RNA-binding, signal transduction-associated protein 1 isoform 1 | -3.34 X 10^+00^ | 3.87 X 10^-09^ |
| TMA7 | translation machinery-associated protein 7 isoform 1 | -3.40 X 10^+00^ | 5.94 X 10^-06^ |
| MED29 | mediator of RNA polymerase II transcription subunit 29 isoform 1 | -3.40 X 10^+00^ | 3.22 X 10^-02^ |
| RBM8A | RNA-binding protein 8A | -3.40 X 10^+00^ | 6.65 X 10^-06^ |
| UBA52 | ubiquitin-60S ribosomal protein L40 isoform X2 | -3.41 X 10^+00^ | 7.87 X 10^-03^ |
| CNBP | cellular nucleic acid-binding protein isoform 6 | -3.43 X 10^+00^ | 1.00 X 10^-09^ |
| FAM8A1 | protein FAM8A1 | -3.46 X 10^+00^ | 1.87 X 10^-02^ |
| RNF103-CHMP3 | RNF103-CHMP3 protein | -3.46 X 10^+00^ | 3.56 X 10^-03^ |
| RPS27L | 40S ribosomal protein S27-like | -3.54 X 10^+00^ | 5.19 X 10^-03^ |
| PALM | paralemmin-1 isoform X1 | -3.57 X 10^+00^ | 5.08 X 10^-03^ |
| RBM41 | RNA-binding protein 41 isoform 4 | -3.57 X 10^+00^ | 1.08 X 10^-04^ |
| YTHDF2 | YTH domain-containing family protein 2 isoform 1 | -3.63 X 10^+00^ | 5.56 X 10^-06^ |
| BCAP31 | B-cell receptor-associated protein 31 isoform a | -3.64 X 10^+00^ | 1.30 X 10^-07^ |
| CHMP1B | charged multivesicular body protein 1b | -3.70 X 10^+00^ | 3.49 X 10^-06^ |
| ABCG4 | ATP-binding cassette sub-family G member 4 isoform b | -3.72 X 10^+00^ | 4.04 X 10^-03^ |
| SERF2 | small EDRK-rich factor 2 isoform a | -3.72 X 10^+00^ | 5.25 X 10^-03^ |
| AHSG | alpha-2-HS-glycoprotein isoform 2 preproprotein | -3.72 X 10^+00^ | 1.23 X 10^-03^ |
| RUSF1 | RUS1 family protein C16orf58 | -3.74 X 10^+00^ | 3.89 X 10^-03^ |
| ATP5MPL | 6.8 kDa mitochondrial proteolipid isoform 2 | -3.76 X 10^+00^ | 6.13 X 10^-03^ |
| IGFBP2 | insulin-like growth factor-binding protein 2 isoform a precursor | -3.78 X 10^+00^ | 4.47 X 10^-04^ |
| MT1G | metallothionein-1G isoform 2 | -3.80 X 10^+00^ | 2.06 X 10^-03^ |
|  | prothymosin alpha isoform X1 | -3.82 X 10^+00^ | 2.56 X 10^-08^ |
| MANBAL | protein MANBAL isoform X1 | -3.84 X 10^+00^ | 1.13 X 10^-02^ |
| ATP8 | ATP synthase F0 subunit 8 (mitochondrion) | -3.84 X 10^+00^ | 6.46 X 10^-05^ |
| HSPA6 | heat shock 70 kDa protein 6 | -3.90 X 10^+00^ | 1.49 X 10^-09^ |
| CCDC43 | coiled-coil domain-containing protein 43 isoform 1 | -3.92 X 10^+00^ | 6.75 X 10^-04^ |
| PARD6G | partitioning defective 6 homolog gamma | -4.01 X 10^+00^ | 9.19 X 10^-04^ |
| RTN1 | reticulon-1 isoform A | -4.01 X 10^+00^ | 2.36 X 10^-07^ |
|  | protein phosphatase 1 regulatory subunit 12A isoform X1 | -4.16 X 10^+00^ | 2.49 X 10^-03^ |
| RRP1 | ribosomal RNA processing protein 1 homolog A | -4.24 X 10^+00^ | 8.30 X 10^-03^ |
| TMSB10 | thymosin beta-10 | -4.29 X 10^+00^ | 2.38 X 10^-10^ |
| ATXN1 | ataxin-1 Alt-ATXN1 | -4.38 X 10^+00^ | 4.47 X 10^-05^ |
| ZNF66 | putative zinc finger protein 66 | -4.57 X 10^+00^ | 5.27 X 10-^04^ |
|  | coiled-coil domain-containing protein 12 isoform X1 | -4.61 X 10^+00^ | 1.47 X 10^-04^ |
| PPP1CC | serine/threonine-protein phosphatase PP1-gamma catalytic subunit isoform X1 | -4.76 X 10^+00^ | 2.08 X 10^-05^ |
