# Supplementary material for: Nicotinic receptor components of amyloid beta 42 proteome regulation in human neural cells
Source: PLoS One. 2022 Aug 12;17(8):e0270479. doi: 10.1371/journal.pone.0270479 (PMC9374227; doi:10.1371/journal.pone.0270479)
Supplement: S1 File — (ZIP) [file pone.0270479.s001.zip › STable3AbBGTX.docx]

**Supplement Table 3: A𝛽_42_/Bgtx Proteome (A𝛽_42_/Bgtx^P^)**

| **gene**  **symbol** | **description** | **log_2_ fold change**  **A𝛽_42_** | **fold change A𝛽_42_**  **p-value** |
| --- | --- | --- | --- |
| BASP1 | brain acid soluble protein 1 | 6.48 X 10^-01^ | 2.19 X 10^-05^ |
| JAGN1 | protein jagunal homolog 1 | 1.01 X 10^+00^ | 4.72 X 10^-03^ |
| MED12 | mediator of RNA polymerase II transcription subunit 12 | 8.67 X 10^-01^ | 1.99 X 10^-02^ |
| TRIM26 | tripartite motif-containing protein 26 isoform X1 | 1.28 X 10^+00^ | 1.30 X 10-^05^ |
| CRB2 | protein crumbs homolog 2 precursor | 8.29 X 10^-01^ | 7.02 X 10^-04^ |
| PTMS | parathymosin isoform 2 | 2.83 X 10^-01^ | 5.70 X 10^-04^ |
| IKBIP | inhibitor of nuclear factor kappa-B kinase-interacting protein isoform 1 | 6.64 X 10^-01^ | 1.39 X 10^-02^ |
| GTF3A | transcription factor IIIA | 7.94 X 10^-01^ | 3.60 X 10^-04^ |
| PALD1 | paladin isoform X1 | 9.48 X 10^-01^ | 3.10 X 10^-03^ |
| PTMA | prothymosin alpha isoform X1 | 3.54 X 10^-01^ | 4.65 X 10^-02^ |
| APPL2 | DCC-interacting protein 13-beta isoform 2 | 1.87 X 10^-01^ | 3.11 X 10^-02^ |
| RSRC2 | arginine/serine-rich coiled-coil protein 2 isoform X1 | 8.86 X 10^-01^ | 1.62 X 10^-02^ |
| SUMO2 | small ubiquitin-related modifier 2 isoform a precursor | 1.93 X 10^-01^ | 6.25 X 10^-03^ |
| ATPIF1 | ATPase inhibitor, mitochondrial isoform 1 precursor | 4.35 X 10^-01^ | 5.94 X 10^-04^ |
| IL12RB2 | interleukin-12 receptor subunit beta-2 isoform X1 | -9.94 X 10^-01^ | 2.29 X 10^-07^ |
| INTS12 | integrator complex subunit 12 | -1.85 X 10^+00^ | 3.44 X 10^-08^ |
| PCTP | phosphatidylcholine transfer protein isoform 4 | 4.83 X 10^-01^ | 1.02 X 10^-02^ |
| LMNB1 | lamin-B1 isoform 1 | 3.33 X 10^-01^ | 1.82 X 10^-03^ |
| PDCD10 | programmed cell death protein 10 isoform X1 | 5.59 X 10^-01^ | 7.09 X 10^-05^ |
| GOLGA4 | golgin subfamily A member 4 isoform X1 | 7.20 X 10^-01^ | 2.18 X 10^-02^ |
| SRSF2 | serine/arginine-rich splicing factor 2 isoform X1 | 2.27 X 10^-01^ | 1.09 X 10^-02^ |
| MAPK1IP1L | MAPK-interacting and spindle-stabilizing protein-like isoform X1 | 8.07 X 10^-01^ | 1.33 X 10^-03^ |
| LAMA3 | laminin subunit alpha-3 isoform X1 | 6.81 X 10^-01^ | 5.90 X 10^-03^ |
| TMPO | thymopoietin isoform beta | 3.36 X 10^-01^ | 2.99 X 10-^05^ |
| SOD2 | superoxide dismutase [Mn], mitochondrial isoform A precursor | 5.98 X 10^-01^ | 6.07 X 10^-04^ |
| CALM2 | calmodulin-2 isoform 1 | 2.03 X 10^-01^ | 3.89 X 10^-03^ |
| TMED2 | transmembrane emp24 domain-containing protein 2 isoform 2 precursor | 5.46 X 10^-01^ | 4.84 X 10^-02^ |
| IRAK1 | interleukin-1 receptor-associated kinase 1 isoform 1 | 6.89 X 10^-01^ | 1.10 X 10^-02^ |
| FEZF2 | fez family zinc finger protein 2 | 4.48 X 10^-01^ | 7.87 X 10^-06^ |
| ARID1A | AT-rich interactive domain-containing protein 1A isoform a | 8.07 X 10^-01^ | 5.78 X 10^-05^ |
| CD59 | CD59 glycoprotein preproprotein | 2.99 X 10^-01^ | 2.45 X 10^-02^ |
| MTMR9 | myotubularin-related protein 9 | 7.50 X 10^-01^ | 2.92 X 10^-02^ |
| GNAI1 | guanine nucleotide-binding protein G(i) subunit alpha-1 isoform 1 | 3.63 X 10^-01^ | 5.56 X 10^-03^ |
| RPS27A | ubiquitin-40S ribosomal protein S27a precursor | 3.10 X 10^-01^ | 5.78 X 10^-06^ |
| FAM49B | protein FAM49B isoform 1 | 3.13 X 10^-01^ | 1.14 X 10^-03^ |
| SLC44A2 | choline transporter-like protein 2 isoform X1 | -9.02 X 10^-01^ | 2.89 X 10^-06^ |
| SNAP29 | synaptosomal-associated protein 29 | -1.04 X 10+00 | 1.73 X 10^-04^ |
| SMARCA4 | transcription activator BRG1 isoform A | 5.87 X 10^-01^ | 9.70 X 10^-15^ |
| GNAI3 | guanine nucleotide-binding protein G(k) subunit alpha | 2.41 X 10^-01^ | 3.80 X 10^-02^ |
| CCNB1 | G2/mitotic-specific cyclin-B1 isoform 1 | 6.05 X 10^-01^ | 2.04 X 10^-02^ |
| FAM118B | protein FAM118B isoform 1 | 1.13 X 10^+00^ | 1.30 X 10^-05^ |
| LGALS8 | galectin-8 isoform a | 1.16 X 10^+00^ | 7.18 X 10^-04^ |
| NONO | non-POU domain-containing octamer-binding protein isoform 1 | 2.55 X 10^-01^ | 2.30 X 10^-04^ |
| TMA7 | translation machinery-associated protein 7 isoform 1 | 3.44 X 10^-01^ | 1.18 X 10^-03^ |
| NDC80 | kinetochore protein NDC80 homolog | 2.61 X 10^-01^ | 2.15 X 10^-02^ |
| YWHAG | 14-3-3 protein gamma | 1.74 X 10^-01^ | 1.54 X 10^-02^ |
| GNAO1 | guanine nucleotide-binding protein G(o) subunit alpha isoform b | 2.56 X 10^-01^ | 2.58 X 10^-02^ |
| PCBD1 | pterin-4-alpha-carbinolamine dehydratase isoform 1 | 7.09 X 10^-01^ | 4.92 X 10^-10^ |
| ATP5F1 | ATP synthase F(0) complex subunit B1, mitochondrial precursor | 3.86 X 10^-01^ | 3.69 X 10^-06^ |
| LRWD1 | leucine-rich repeat and WD repeat-containing protein 1 isoform 1 | 4.23 X 10^-01^ | 1.35 X 10^-02^ |
| TRAPPC2L | trafficking protein particle complex subunit 2-like protein isoform 1 | 5.44 X 10^-01^ | 2.64 X 10^-05^ |
| CNTFR | ciliary neurotrophic factor receptor subunit alpha isoform X1 | 2.92 X 10^-01^ | 2.36 X 10^-02^ |
| SMARCB1 | SWI/SNF-related matrix-associated actin-dependent regulator of chromatin subfamily B member 1 isoform X1 | 3.63 X 10^-01^ | 2.77 X 10^-02^ |
| SMARCC2 | SWI/SNF complex subunit SMARCC2 isoform a | 3.35 X 10^-01^ | 1.94 X 10^-04^ |
| TUBAL3 | tubulin alpha chain-like 3 isoform 1 | -7.76 X 10^-01^ | 1.94 X 10^-04^ |
| DNMT1 | DNA (cytosine-5)-methyltransferase 1 isoform a | 2.57 X 10^-01^ | 2.44 X 10^-02^ |
| PHB | prohibitin isoform 1 | 2.42 X 10^-01^ | 4.74 X 10^-04^ |
| RPN2 | dolichyl-diphosphooligosaccharide--protein glycosyltransferase subunit 2 isoform 7 precursor | 2.76 X 10^-01^ | 2.60 X 10^-04^ |
| AK6 | adenylate kinase isoenzyme 6 isoform b | 5.37 X 10^-01^ | 4.54 X 10^-04^ |
| PPP1CC | serine/threonine-protein phosphatase PP1-gamma catalytic subunit isoform X1 | 9.88 X 10^-01^ | 4.18 X 10^-07^ |
| NEDD8 | NEDD8 precursor | 1.53 X 10^-01^ | 3.30 X 10^-02^ |
| SDHD | succinate dehydrogenase [ubiquinone] cytochrome b small subunit, mitochondrial isoform a precursor | 8.57 X 10^-01^ | 4.43 X 10^-03^ |
| TMEM161A | transmembrane protein 161A isoform 1 precursor | -8.62 X 10^-01^ | 5.94 X 10^-04^ |
| PHB2 | prohibitin-2 isoform 1 | 3.12 X 10^-01^ | 5.11 X 10^-06^ |
| GDAP1 | ganglioside-induced differentiation-associated protein 1 isoform a | 2.17 X 10^-01^ | 2.49 X 10^-02^ |
| MYO1B | unconventional myosin-Ib isoform 1 | 3.56 X 10^-01^ | 1.76 X 10^-07^ |
| RHEB | GTP-binding protein Rheb | 2.07 X 10^-01^ | 2.58 X 10^-02^ |
| HMG20B | SWI/SNF-related matrix-associated actin-dependent regulator of chromatin subfamily E member 1-related | -5.82 X 10^-01^ | 1.94 X 10^-03^ |
| PSMD12 | 26S proteasome non-ATPase regulatory subunit 12 isoform 1 | 1.62 X 10^-01^ | 2.42 X 10^-02^ |
| NUMBL | numb-like protein isoform a | -2.78 X 10^+00^ | 4.47 X 10^-16^ |
| TBRG4 | FAST kinase domain-containing protein 4 isoform 3 | 2.40 X 10^-01^ | 1.50 X 10^-02^ |
| GNAS | protein GNAS isoform X1 | 1.63 X 10^-01^ | 2.76 X 10^-02^ |
| GNAI2 | guanine nucleotide-binding protein G(i) subunit alpha-2 isoform 1 | 1.61 X 10^-01^ | 3.74 X 10^-02^ |
| MLLT1 | protein ENL isoform X1 | 3.53 X 10^-01^ | 2.66 X 10^-02^ |
| TBR1 | T-box brain protein 1 | -8.29 X 10^-01^ | 7.06 X 10^-06^ |
| PSMB5 | proteasome subunit beta type-5 isoform 1 | 1.52 X 10^-01^ | 3.44 X 10^-02^ |
| GPR142 | probable G-protein coupled receptor 142 isoform a | 3.14 X 10^-01^ | 2.70 X 10^-02^ |
| SLC25A11 | mitochondrial 2-oxoglutarate/malate carrier protein isoform 1 | 4.45 X 10^-01^ | 1.83 X 10^-04^ |
| SREK1 | splicing regulatory glutamine/lysine-rich protein 1 isoform a | 4.46 X 10^-01^ | 1.70 X 10-^02^ |
| ENO3 | beta-enolase isoform X1 | 1.78 X 10^-01^ | 1.30 X 10^-02^ |
| RPS15 | 40S ribosomal protein S15 isoform 1 | -3.73 X 10^-01^ | 1.56 X 10^-03^ |
| ATP5L | ATP synthase subunit g, mitochondrial | 2.22 X 10^-01^ | 1.01 X 10^-02^ |
| PFDN2 | prefoldin subunit 2 | 2.00 X 10^-01^ | 4.90 X 10^-03^ |
| GSTA4 | glutathione S-transferase A4 | 3.98 X 10^-01^ | 4.64 X 10^-02^ |
| SLC25A3 | phosphate carrier protein, mitochondrial isoform a precursor | 2.15 X 10^-01^ | 2.21 X 10^-03^ |
| MAOA | amine oxidase [flavin-containing] A isoform 1 | 1.95 X 10^-01^ | 5.49 X 10^-03^ |
| MYO1C | unconventional myosin-Ic isoform a | 2.76 X 10^-01^ | 3.64 X 10^-03^ |
| OGFOD1 | prolyl 3-hydroxylase OGFOD1 isoform 4 | 4.54 X 10^-01^ | 1.64 X 10^-02^ |
| TMEM11 | transmembrane protein 11, mitochondrial | 4.17 X 10^-01^ | 3.11 X 10^-02^ |
| RAB6A | ras-related protein Rab-6A isoform a | 2.44 X 10^-01^ | 9.15 X 10^-03^ |
| SATB1 | DNA-binding protein SATB1 isoform 2 | 4.48 X 10^-01^ | 9.53 X 10^-03^ |
| SLC25A5 | ADP/ATP translocase 2 | 1.79 X 10^-01^ | 1.24 X 10^-02^ |
| FLOT1 | flotillin-1 isoform X1 | 2.44 X 10^-01^ | 3.80 X 10^-02^ |
| EIF4G2 | eukaryotic translation initiation factor 4 gamma 2 isoform 1 | 1.70 X 10^-01^ | 2.66 X 10^-02^ |
| BOLA2 | bolA-like protein 2 isoform 1 | 1.48 X 10^-01^ | 4.19 X 10^-02^ |
| ZNF181 | zinc finger protein 181 isoform X1 | 3.31 X 10^-01^ | 8.21 X 10^-03^ |
| GGH | gamma-glutamyl hydrolase precursor | 2.57 X 10^-01^ | 1.45 X 10^-02^ |
| CDC14B | dual specificity protein phosphatase CDC14B isoform X9 | 5.59 X 10^-01^ | 2.90 X 10^-05^ |
| FAHD1 | acylpyruvase FAHD1, mitochondrial isoform 1 | 2.96 X 10^-01^ | 2.20 X 10^-02^ |
| ISYNA1 | inositol-3-phosphate synthase 1 isoform X3 | 1.92 X 10^-01^ | 2.60 X 10^-02^ |
| ACTC1 | actin, alpha cardiac muscle 1 precursor | 1.49 X 10^-01^ | 3.88 X 10^-02^ |
| CDK7 | cyclin-dependent kinase 7 isoform 1 | -8.57 X 10^-01^ | 3.75 X 10^-02^ |
| KRT10 | keratin, type I cytoskeletal 10 isoform X1 | -1.77 X 10^+00^ | 4.47 X 10^-16^ |
| C17orf49 | chromatin complexes subunit BAP18 isoform 1 | 5.66 X 10^-01^ | 4.18 X 10^-02^ |
| SRP9 | signal recognition particle 9 kDa protein isoform 2 | -2.99 X 10^-01^ | 2.50 X 10^-02^ |
| RNLS | renalase isoform 1 precursor | 2.92 X 10^-01^ | 2.49 X 10^-02^ |
| MGST3 | microsomal glutathione S-transferase 3 isoform X1 | 2.41 X 10^-01^ | 4.09 X 10^-02^ |
| THEM6 | protein THEM6 precursor | 1.87 X 10^-01^ | 9.01 X 10^-03^ |
| VKORC1L1 | vitamin K epoxide reductase complex subunit 1-like protein 1 isoform 1 | -4.78 X 10^-01^ | 3.23 X 10^-02^ |
| GGT7 | glutathione hydrolase 7 isoform 1 | -5.57 X 10^+00^ | 4.47 X 10^-16^ |
| UPF3B | regulator of nonsense transcripts 3B isoform X1 | -6.76 X 10^-01^ | 1.43 X 10^-02^ |
| KRT2 | keratin, type II cytoskeletal 2 epidermal | -1.49 X 10^+00^ | 4.47 X 10^-16^ |
| UROD | uroporphyrinogen decarboxylase | -3.57 X 10^-01^ | 7.92 X 10^-03^ |
| HDAC6 | histone deacetylase 6 isoform a | -3.06 X 10^-01^ | 2.21 X 10^-02^ |
| PPP2R2D | serine/threonine-protein phosphatase 2A 55 kDa regulatory subunit B delta isoform isoform a | 1.06 X 10^+00^ | 7.24 X 10^-06^ |
| NUTF2 | nuclear transport factor 2 | -3.92 X 10^-01^ | 1.93 X 10^-04^ |
| BCL7C | B-cell CLL/lymphoma 7 protein family member C isoform X1 | -6.21 X 10^-01^ | 2.20 X 10^-03^ |
| TMEM106B | transmembrane protein 106B | -6.48 X 10^-01^ | 3.53 X 10^-02^ |
| RPS24 | 40S ribosomal protein S24 isoform d | -3.09 X 10^-01^ | 1.39 X 10^-02^ |
| SF3B14 | splicing factor 3B subunit 6 | -4.09 X 10^-01^ | 2.49 X 10^-02^ |
| NOL4 | nucleolar protein 4 isoform X1 | -6.60 X 10^-01^ | 1.83 X 10^-04^ |
| RABGEF1 | rab5 GDP/GTP exchange factor isoform b | 9.96 X 10^-01^ | 4.32 X 10^-03^ |
| BCAT2 | branched-chain-amino-acid aminotransferase, mitochondrial isoform a precursor | 8.58 X 10^-01^ | 2.49 X 10^-02^ |
| SSSCA1 | Sjoegren syndrome/scleroderma autoantigen 1 isoform 1 | -6.46 X 10^-01^ | 2.49 X 10^-02^ |
| TDP2 | tyrosyl-DNA phosphodiesterase 2 | -9.74 X 10^-01^ | 2.65 X 10^-03^ |
| LONP2 | lon protease homolog 2, peroxisomal isoform 3 | 7.84 X 10^-01^ | 2.90 X 10^-05^ |
| CCDC66 | coiled-coil domain-containing protein 66 isoform 3 | -9.94 X 10^-01^ | 5.70 X 10^-04^ |
| CDIPT | CDP-diacylglycerol--inositol 3-phosphatidyltransferase isoform X1 | 1.16 X 10^+00^ | 6.50 X 10^-04^ |
| SCOC | short coiled-coil protein isoform 1 | -7.39 X 10^-01^ | 2.54 X 10^-05^ |
| FAM186B | protein FAM186B isoform X1 | -6.80 X 10-01 | 2.03 X 10^-05^ |
| MRPS10 | 28S ribosomal protein S10, mitochondrial isoform X1 | 5.74 X 10-01 | 3.11 X 10^-02^ |
| SRGAP1 | SLIT-ROBO Rho GTPase-activating protein 1 isoform 1 | 7.32 X 10-01 | 5.49 X 10^-03^ |
| G3BP2 | ras GTPase-activating protein-binding protein 2 isoform X1 | -3.26 X 10-01 | 2.87 X 10^-02^ |
| RPL37A | 60S ribosomal protein L37a | -4.40 X 10-01 | 9.70 X 10^-06^ |
| LUC7L | putative RNA-binding protein Luc7-like 1 isoform b | -3.73 X 10-01 | 2.79 X 10^-02^ |
| HMGN2 | non-histone chromosomal protein HMG-17 | 5.98 X 10-01 | 2.46 X 10^-04^ |
| ENSA | alpha-endosulfine isoform 1 | -6.62 X 10-01 | 2.36 X 10^-03^ |
| COA3 | cytochrome c oxidase assembly factor 3 homolog, mitochondrial | -8.65 X 10-01 | 4.17 X 10^-03^ |
| RPL39 | 60S ribosomal protein L39 | -2.97 X 10-01 | 2.30 X 10^-02^ |
| FAM136A | protein FAM136A isoform 2 | -5.15 X 10-01 | 1.04 X 10^-03^ |
| HNRNPM | heterogeneous nuclear ribonucleoprotein M isoform a | -3.64 X 10-01 | 8.46 X 10^-04^ |
| BAG1 | BAG family molecular chaperone regulator 1 isoform BAG-1L | 1.50 X 10+00 | 1.13 X 10^-11^ |
| PRKCI | protein kinase C iota type | -5.86 X 10-01 | 2.12 X 10^-02^ |
| AP1S1 | AP-1 complex subunit sigma-1A | -6.76 X 10-01 | 1.09 X 10^-02^ |
| RBM3 | RNA-binding protein 3 | -4.82 X 10-01 | 6.84 X 10^-03^ |
| SUPT16H | FACT complex subunit SPT16 | -3.79 X 10^-01^ | 1.53 X 10^-03^ |
| MED23 | mediator of RNA polymerase II transcription subunit 23 isoform X1 | -1.10 X 10^+00^ | 3.81 X 10^-03^ |
| COX6C | cytochrome c oxidase subunit 6C isoform X1 | -6.26 X 10^-01^ | 9.85 X 10^-03^ |
| HEATR5A | HEAT repeat-containing protein 5A | 1.22 X 10^+00^ | 1.33 X 10^-06^ |
| UBTF | nucleolar transcription factor 1 isoform X1 | -4.05 X 10^-01^ | 1.27 X 10^-03^ |
| ERH | enhancer of rudimentary homolog | -3.06 X 10^-01^ | 1.92 X 10^-02^ |
|  | FACT complex subunit SSRP1 isoform X1 | -4.50 X 10^-01^ | 2.11 X 10^-06^ |
| MAGI3 | membrane-associated guanylate kinase, WW and PDZ domain-containing protein 3 isoform 1 | -1.43 X 10^+00^ | 5.17 X 10^-09^ |
| DPM3 | dolichol-phosphate mannosyltransferase subunit 3 isoform 1 | 1.03 X 10^+00^ | 3.42 X 10^-10^ |
| UMODL1 | uromodulin-like 1 isoform 2 precursor | 7.32 X 10^-01^ | 7.95 X 10^-04^ |
| BCLAF1 | bcl-2-associated transcription factor 1 isoform 1 | -3.57 X 10^-01^ | 7.92 X 10^-03^ |
| CCNK | cyclin-K | -1.13 X 10^+00^ | 1.20 X 10^-03^ |
| ERI3 | ERI1 exoribonuclease 3 isoform 1 | -9.86 X 10^-01^ | 2.71 X 10^-02^ |
| TAB3 | TGF-beta-activated kinase 1 and MAP3K7-binding protein 3 isoform X1 | -6.51 X 10^-01^ | 1.68 X 10^-03^ |
| SNRPE | small nuclear ribonucleoprotein E isoform 1 | -3.88 X 10^-01^ | 5.11 X 10^-04^ |
| SURF4 | surfeit locus protein 4 isoform 1 | -6.17 X 10^-01^ | 6.33 X 10^-04^ |
| MAP7 | ensconsin isoform X1 | -7.06 X 10^-01^ | 2.56 X 10^-02^ |
| SLTM | SAFB-like transcription modulator isoform X1 | -7.61 X 10^-01^ | 1.62 X 10^-02^ |
| LARP4 | la-related protein 4 isoform X1 | -1.46 X 10^+00^ | 1.76 X 10^-07^ |
| POU5F2 | POU domain, class 5, transcription factor 2 | -1.03 X 10^+00^ | 1.78 X 10^-02^ |
| NDRG4 | protein NDRG4 isoform X7 | -8.92 X 10^-01^ | 2.47 X 10^-04^ |
| PCBP2 | poly(rC)-binding protein 2 isoform f | -1.08 X 10^+00^ | 9.63 X 10^-03^ |
| ITPK1 | inositol-tetrakisphosphate 1-kinase isoform X1 | -1.00 X 10^+00^ | 1.14 X 10^-03^ |
| ERI1 | 3'-5' exoribonuclease 1 isoform X1 | -6.76 X 10^-01^ | 7.13 X 10^-03^ |
| TUBB8 | tubulin beta-8 chain | -2.92 X 10^+00^ | 4.47 X 10-^16^ |
| LOC102724985 | pyridoxal-dependent decarboxylase domain-containing protein 1 isoform X1 | -7.93 X 10^-01^ | 2.30 X 10^-02^ |
| ZNF232 | zinc finger protein 232 isoform a | -1.11 X 10^+00^ | 1.21 X 10^-04^ |
| KRT1 | keratin, type II cytoskeletal 1 | -5.08 X 10^-01^ | 1.95 X 10^-02^ |
