# Supplementary material for: Nicotinic receptor components of amyloid beta 42 proteome regulation in human neural cells
Source: PLoS One. 2022 Aug 12;17(8):e0270479. doi: 10.1371/journal.pone.0270479 (PMC9374227; doi:10.1371/journal.pone.0270479)
Supplement: S1 File — (ZIP) [file pone.0270479.s001.zip › STable4deltaP.docx]

**Supplement Table 4: ΔP Dataset**

| gene symbol | description | log_2_ fold change  A𝛽_42_ | fold change A𝛽_42_  p-value | log_2_ fold change  A𝛽_42_+ Bgtx | fold change  A𝛽_42_+ Bgtx  p-value |
| --- | --- | --- | --- | --- | --- |
| ALS2 | alsin isoform X1 | -2.22 X 10^00^ | 5.25 X 10^-16^ | -5.23 X 10^-01^ | 2.50 X 10^-01^ |
| C9orf84 | uncharacterized protein C9orf84 isoform X1 | -9.05 X 10^-01^ | 2.48 X 10^-03^ | 4.45 X 10^-01^ | 1.61 X 10^-01^ |
| RBM41 | RNA-binding protein 41 isoform 4 | -8.52 X 10^-01^ | 1.21 X 10^-04^ | 3.73 X 10^-01^ | 1.05 X 10^-01^ |
| NME1 | nucleoside diphosphate kinase A isoform a | -6.99 X 10^-01^ | 3.86 X 10^-02^ | 4.09 X 10^-01^ | 2.18 X 10^-01^ |
| CCDC33 | coiled-coil domain-containing protein 33 isoform X1 | -1.25 X 10^+00^ | 4.89 X 10^-04^ | -2.09 X 10^-01^ | 9.32 X 10^-01^ |
| TMSB4X | thymosin beta-4 | -9.30 X 10^-01^ | 1.32 X 10^-04^ | -5.89 X 10^-02^ | 9.99 X 10^-01^ |
| MKRN3 | probable E3 ubiquitin-protein ligase makorin-3 | -7.86 X 10^-01^ | 8.22 X 10^-04^ | -1.86 X 10^-01^ | 9.12 X 10^-01^ |
| ADAM22 | disintegrin and metalloproteinase domain-containing protein 22 isoform X1 | -7.56 X 10^-01^ | 2.80 X 10^-02^ | -2.38 X 10^-01^ | 8.89 X 10^-01^ |
| BCAP31 | B-cell receptor-associated protein 31 isoform a | -5.40 X 10^-01^ | 3.23 X 10^-05^ | 1.39 X 10^-01^ | 2.43 X 10^-01^ |
| MINOS1 | MICOS complex subunit MIC10 isoform c | -8.73 X 10^-01^ | 8.82 X 10^-04^ | -4.27 X 10^-01^ | 5.19 X 10^-01^ |
| TMSB10 | thymosin beta-10 | -4.34 X 10^-01^ | 2.43 X 10^-04^ | -1.60 X 10^-02^ | 8.97 X 10^-01^ |
|  | granulins isoform X1 | -4.98 X 10^-01^ | 1.39 X 10^-02^ | -9.54 X 10^-02^ | 9.99 X 10^-01^ |
| NUCKS1 | nuclear ubiquitous casein and cyclin-dependent kinase substrate 1 | -4.74 X 10^-01^ | 1.31 X 10^-05^ | -8.47 X 10^-02^ | 9.85 X 10^-01^ |
| CALU | calumenin isoform c precursor | -3.20 X 10^-01^ | 4.48 X 10^-02^ | -7.23 X 10^-03^ | 8.88 X 10^-01^ |
| CNBP | cellular nucleic acid-binding protein isoform 3 | -3.44 X 10^-01^ | 1.45 X 10^-02^ | -9.08 X 10^-02^ | 9.77 X 10^-01^ |
| POLR1D | DNA-directed RNA polymerases I and III subunit RPAC2 isoform 1 | -7.91 X 10^-01^ | 2.91 X 10^-02^ | -5.19 X 10^-01^ | 4.31 X 10^-01^ |
| RPS28 | 40S ribosomal protein S28 | -3.51 X 10^-01^ | 2.80 X 10^-02^ | -8.01 X 10^-02^ | 9.92 X 10^-01^ |
| YBX1 | nuclease-sensitive element-binding protein 1 | -4.92 X 10^-01^ | 5.60 X 10^-06^ | -1.03 X 10^-01^ | 9.32 X 10^-01^ |
| VAV2 | guanine nucleotide exchange factor VAV2 isoform X1 | -4.52 X 10^-01^ | 2.80 X 10^-02^ | -1.89 X 10^-01^ | 8.89 X 10^-01^ |
| BID | BH3-interacting domain death agonist isoform 1 | -4.02 X 10^-01^ | 2.80 X 10^-02^ | -2.43 X 10^-01^ | 4.67 X 10^-01^ |
| CLTA | clathrin light chain A isoform a | -4.86 X 10^-01^ | 1.46 X 10^-05^ | -2.19 X 10^-01^ | 3.74 X 10^-01^ |
| CTSB | cathepsin B isoform X1 | -3.24 X 10^-01^ | 3.54 X 10^-02^ | -1.81 X 10^-01^ | 6.20 X 10^-01^ |
| CKS1B | cyclin-dependent kinases regulatory subunit 1 | -4.60 X 10^-01^ | 1.34 X 10^-03^ | -3.00 X 10^-01^ | 1.67 X 10^-01^ |
| MAP1A | microtubule-associated protein 1A | -8.49 X 10^-01^ | 1.98 X 10^-02^ | -4.82 X 10^-01^ | 5.95 X 10^-01^ |
| CHCHD2 | coiled-coil-helix-coiled-coil-helix domain-containing protein 2 precursor isoform 1 | -3.60 X 10^-01^ | 2.23 X 10^-02^ | -2.21 X 10^-01^ | 4.79 X 10^-01^ |
| PPIG | peptidyl-prolyl cis-trans isomerase G | 4.75 X 10^-01^ | 1.88 X 10^-02^ | 2.05 X 10^-01^ | 6.07 X 10^-01^ |
| COA4 | cytochrome c oxidase assembly factor 4 homolog, mitochondrial isoform X1 | 1.78 X 10^+00^ | 5.25 X 10^-16^ | 7.18 X 10^-02^ | 8.85 X 10^-01^ |
| MIF | macrophage migration inhibitory factor | -5.73 X 10^-01^ | 1.14 X 10^-08^ | -1.16 X 10^-01^ | 8.97 X 10^-01^ |
| AKT3 | RAC-gamma serine/threonine-protein kinase isoform X1 | 2.58 X 10^-01^ | 2.44 X 10^-02^ | 1.20 X 10^-01^ | 4.68 X 10^-01^ |
| CUTA | protein CutA isoform 1 | 2.04 X 10^-01^ | 2.91 X 10^-02^ | 9.63 X 10^-02^ | 5.34 X 10^-01^ |
| PPIE | peptidyl-prolyl cis-trans isomerase E isoform 4 | 4.89 X 10^-01^ | 2.50 X 10^-02^ | 4.85 X 10^-01^ | 7.09 X 10^-02^ |
| NEDD8-MDP1 | NEDD8-MDP1 protein | -3.33 X 10^-01^ | 4.21 X 10^-02^ | -1.52 X 10^-01^ | 7.79 X 10^-01^ |
| ECI1 | enoyl-CoA delta isomerase 1, mitochondrial isoform 1 precursor | -3.64 X 10^-01^ | 6.93 X 10^-03^ | -1.91 X 10^-01^ | 5.35 X 10^-01^ |
| SFXN3 | sideroflexin-3 | 1.95 X 10^-01^ | 9.49 X 10^-03^ | 1.75 X 10^-01^ | 8.72 X 10^-02^ |
| UQCRC2 | cytochrome b-c1 complex subunit 2, mitochondrial precursor | 2.28 X 10^-01^ | 1.14 X 10^-02^ | 1.52 X 10^-01^ | 2.53 X 10^-01^ |
| LONP1 | lon protease homolog, mitochondrial isoform 1 precursor | 1.43 X 10^-01^ | 2.92 X 10^-02^ | 7.45 X 10^-02^ | 3.77 X 10^-01^ |
| BORCS7 | BLOC-1-related complex subunit 7 | 1.81 X 10^-01^ | 2.56 X 10^-02^ | 1.30 X 10^-01^ | 2.73 X 10^-01^ |
| IMMT | MICOS complex subunit MIC60 isoform X1 | 1.40 X 10^-01^ | 4.21 X 10^-02^ | 9.76 X 10^-02^ | 2.78 X 10^-01^ |
| SLC25A13 | calcium-binding mitochondrial carrier protein Aralar2 isoform X1 | 1.90 X 10^-01^ | 4.63 X 10^-03^ | 9.63 X 10^-02^ | 2.48 X 10^-01^ |
| ANKHD1 | ANKHD1-EIF4EBP3 protein | 3.71 X 10^-01^ | 3.12 X 10^-02^ | 2.64 X 10^-01^ | 1.71 X 10^-01^ |
| COPS7B | COP9 signalosome complex subunit 7b isoform b | 2.74 X 10^-01^ | 2.73 X 10^-03^ | 1.69 X 10^-01^ | 2.10 X 10^-01^ |
| HACE1 | E3 ubiquitin-protein ligase HACE1 isoform a | 7.71 X 10^-01^ | 1.09 X 10^-02^ | 6.60 X 10^-01^ | 7.08 X 10^-02^ |
| DNAJA3 | dnaJ homolog subfamily A member 3, mitochondrial isoform 1 | 5.03 X 10^-01^ | 2.46 X 10^-04^ | 1.72 X 10^-01^ | 4.12 X 10^-01^ |
| TRMT2A | tRNA (uracil-5-)-methyltransferase homolog A isoform c | 4.59 X 10^-01^ | 2.55 X 10^-02^ | 4.68 X 10^-01^ | 7.04 X 10-^02^ |
| CREBBP | CREB-binding protein isoform a | -1.11 X 10^+00^ | 4.78 X 10^-10^ | -3.90 X 10-^01^ | 4.30 X 10^-01^ |
| PAK4 | serine/threonine-protein kinase PAK 4 isoform X1 | 3.81 X 10-^01^ | 2.61 X 10^-03^ | 1.76 X 10^-01^ | 2.67 X 10^-01^ |
| RPL9 | 60S ribosomal protein L9 | 1.76 X 10^-01^ | 6.85 X 10^-03^ | 4.68 X 10^-02^ | 5.64 X 10^-01^ |
| CASP3 | caspase-3 isoform a preproprotein | 4.68 X 10^-01^ | 5.47 X 10^-08^ | 1.67 X 10^-01^ | 2.02 X 10^-01^ |
| DKC1 | H/ACA ribonucleoprotein complex subunit 4 isoform 1 | 2.49 X 10^-01^ | 4.11 X 10^-02^ | 6.76 X 10^-02^ | 7.07 X 10^-01^ |
| CSTF1 | cleavage stimulation factor subunit 1 | 2.86 X 10^-01^ | 1.46 X 10^-02^ | 1.78 X 10^-01^ | 2.68 X 10^-01^ |
| RBM6 | RNA-binding protein 6 isoform 1 | 7.98 X 10^-01^ | 1.21 X 10^-02^ | 6.14 X 10^-01^ | 1.10 X 10^-01^ |
| DIP2B | disco-interacting protein 2 homolog B | 2.93 X 10^-01^ | 2.38 X 10^-02^ | 1.34 X 10^-01^ | 4.61 X 10^-01^ |
| MTCH2 | mitochondrial carrier homolog 2 isoform 1x | 2.50 X 10-^01^ | 4.35 X 10^-03^ | -1.16 X 10^-02^ | 9.28 X 10^-01^ |
| VDAC1 | voltage-dependent anion-selective channel protein 1 isoform X1 | 2.68 X 10^-01^ | 4.40 X 10^-05^ | 4.12 X 10^-02^ | 6.13 X 10^-01^ |
| VDAC3 | voltage-dependent anion-selective channel protein 3 isoform 2 | 2.36 X 10^-01^ | 3.45 X 10^-04^ | -1.44 X 10^-03^ | 8.84 X 10^-01^ |
| DNAJC11 | dnaJ homolog subfamily C member 11 | 3.79 X 10^-01^ | 7.96 X 10^-03^ | 1.01 X 10^-02^ | 8.92 X 10^-01^ |
| CNOT4 | CCR4-NOT transcription complex subunit 4 isoform f | 6.03 X 10^-01^ | 2.79 X 10^-02^ | 3.57 X 10^-01^ | 4.48 X 10^-01^ |
| VDAC2 | voltage-dependent anion-selective channel protein 2 isoform 1 | 2.23 X 10^-01^ | 5.88 X 10^-04^ | -1.45 X 10^-02^ | 8.97 X 10^-01^ |
| NEFM | neurofilament medium polypeptide isoform 1 | 2.79 X 10^-01^ | 2.63 X 10^-02^ | 0.00 X 10^+00^ | 8.97 X 10^-01^ |
| EPB41L4A | band 4.1-like protein 4A isoform 1 | 5.15 X 10^-01^ | 3.78 X 10^-03^ | 2.61 X 10^-01^ | 3.26 X 10^-01^ |
| AGPAT4 | 1-acyl-sn-glycerol-3-phosphate acyltransferase delta | 9.83 X 10^-01^ | 9.57 X 10^-04^ | 6.85 X 10^-01^ | 9.66 X 10^-02^ |
| FAM188A | ubiquitin carboxyl-terminal hydrolase MINDY-3 isoform a | 7.47 X 10^-01^ | 1.55 X 10^-04^ | 4.11 X 10^-01^ | 1.78 X 10^-01^ |
| FAM45A | protein FAM45A isoform 1 | 1.12 X 10^+00^ | 2.90 X 10^-07^ | 4.95 X 10^-01^ | 1.42 X 10^-01^ |
| FBXO18 | F-box DNA helicase 1 isoform X1 | 4.67 X 10^-01^ | 1.83 X 10^-03^ | 8.00 X 10^-02^ | 7.86 X 10-^01^ |
| TANGO6 | transport and Golgi organization protein 6 homolog | 9.40 X 10^-01^ | 8.22 X 10^-03^ | 5.60 X 10^-01^ | 2.67 X 10^-01^ |
| VPS29 | vacuolar protein sorting-associated protein 29 isoform 1 | 5.41 X 10^-01^ | 7.22 X 10^-04^ | -3.46 X 10^-01^ | 5.35 X 10^-01^ |
| BANF1 | barrier-to-autointegration factor isoform X1 | 7.33 X 10^-01^ | 1.28 X 10^-09^ | 5.24 X 10^-02^ | 8.46 X 10^-01^ |
| HAUS8 | HAUS augmin-like complex subunit 8 isoform a | 6.62 X 10^-01^ | 1.18 X 10^-03^ | 9.76 X 10^-02^ | 8.92 X 10^-01^ |
| CUL4A | cullin-4A isoform 1 | 5.41 X 10^-01^ | 2.80 X 10^-02^ | -4.98 X 10^-01^ | 4.30 X 10^-01^ |
| BASP1 | brain acid soluble protein 1 | -6.57 X 10^-01^ | 2.00 X 10^-03^ | 6.48 X 10^-01^ | 2.19 X 10^-05^ |
| APPL2 | DCC-interacting protein 13-beta isoform 2 | -3.66 X 10^-01^ | 1.01 X 10^-02^ | 1.87 X 10^-01^ | 3.11 X 10^-02^ |
| SUMO2 | small ubiquitin-related modifier 2 isoform a precursor | -3.20 X 10^-01^ | 4.89 X 10^-02^ | 1.93 X 10^-01^ | 6.25 X 10^-03^ |
