# Supplementary material for: Nicotinic receptor components of amyloid beta 42 proteome regulation in human neural cells
Source: PLoS One. 2022 Aug 12;17(8):e0270479. doi: 10.1371/journal.pone.0270479 (PMC9374227; doi:10.1371/journal.pone.0270479)
Supplement: S1 File — (ZIP) [file pone.0270479.s001.zip › SupplementFigl 1.pptx]

## Slide 1
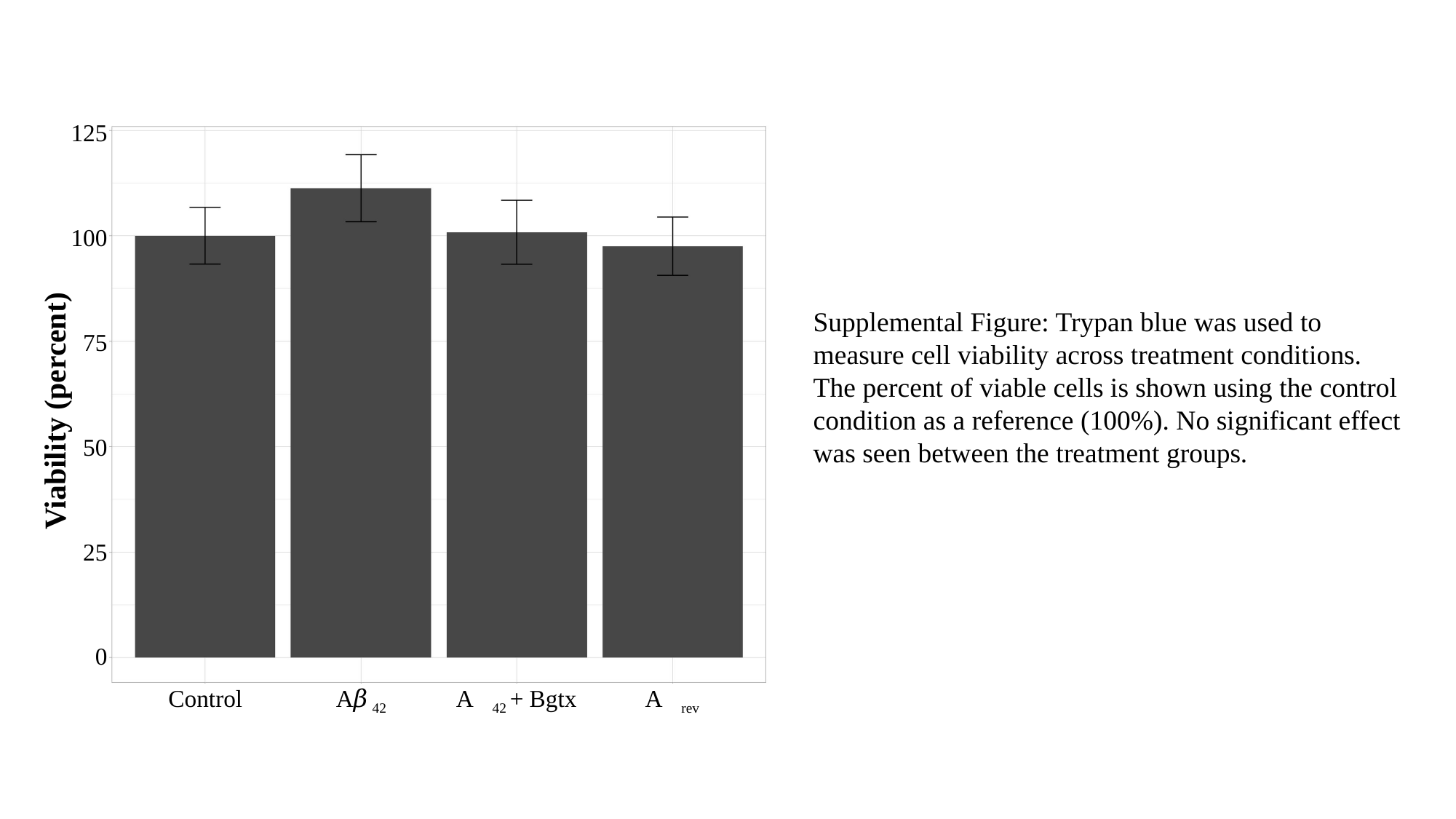

125
100
Supplemental Figure: Trypan blue was used to measure cell viability across treatment conditions. The percent of viable cells is shown using the control condition as a reference (100%). No significant effect was seen between the treatment groups.
75
Viability (percent)
50
25
0
Control
A𝛽42
A𝛽42 + Bgtx
A𝛽rev
